# Two turtles with soft tissue preservation from the platy limestones of Germany provide evidence for marine flipper adaptations in Late Jurassic thalassochelydians

**DOI:** 10.1371/journal.pone.0252355

**Published:** 2021-06-03

**Authors:** Walter G. Joyce, Matthias Mäuser, Serjoscha W. Evers

**Affiliations:** 1 Departement für Geowissenschaften, Universität Freiburg, Freiburg, Switzerland; 2 Staatliche Naturwissenschaftliche Sammlungen Bayerns, Naturkunde-Museum Bamberg, Bamberg, Germany; Royal Belgian Institute of Natural Sciences, BELGIUM

## Abstract

Late Jurassic deposits across Europe have yielded a rich fauna of extinct turtles. Although many of these turtles are recovered from marine deposits, it is unclear which of these taxa are habitually marine and which may be riverine species washed into nearby basins, as adaptations to open marine conditions are yet to be found. Two new fossils from the Late Jurassic of Germany provide unusually strong evidence for open marine adaptations. The first specimen is a partial shell and articulated hind limb from the Late Jurassic (early Tithonian) platy limestones of Schernfeld near Eichstätt, which preserves the integument of the hind limb as an imprint. The skin is fully covered by flat, polygonal scales, which stiffen the pes into a paddle. Although taxonomic attribution is not possible, similarities are apparent with *Thalassemys*. The second specimen is a large, articulated skeleton with hypertrophied limbs referable to *Thalassemys bruntrutana* from the Late Jurassic (early Late Kimmeridgian) platy limestone of Wattendorf, near Bamberg. Even though the skin is preserved as a phosphatic film, the scales are not preserved. This specimen can nevertheless be inferred to have had paddles stiffened by scales based on the pose in which they are preserved, the presence of epibionts between the digits, and by full morphological correspondence to the specimen from Schernfeld. An analysis of scalation in extant turtles demonstrated that elongate flippers stiffed by scales are a marine adaptation, in contrast to the elongate but flexible flippers of riverine turtles. Phylogenetic analysis suggests that *Thalassemys bruntrutana* is referable to the mostly Late Jurassic turtle clade *Thalassochelydia*. The marine adapted flippers of this taxon therefore evolved convergently with those of later clades of marine turtles. Although thalassochelydian fossils are restricted to Europe, with one notable exception from Argentina, their open marine adaptations combined with the interconnectivity of Jurassic oceans predict that the clade must have been even more wide-spread during that time.

## Introduction

A rich assemblage of turtles has been recovered from Late Jurassic platy limestone deposits (= plattenkalks) across France and Germany over the course of the last two centuries (see [1] for recent summary). Although these deposits are universally regarded as marine, the rich associated fauna and flora consisting of vascular plants, insects, lepidosaurs, and small dinosaurs, in addition to marine invertebrates and vertebrates and volant animals such as pterosaurs and early birds, universally suggest relatively close proximity to land, either in the form of small, atoll-like islands that may have dotted the landscape in the immediate vicinity of the basins where the platy limestones were deposited, or larger landmasses, such as the Bohemian, Central, or Rhenish massifs, which likely emerged from the oceans close by [2–5]. A large spectrum of possible habitats was therefore available for the turtles at that time, ranging from the open ocean, reefs, and coastal marshes to freshwater rivers, swamps, and ponds.

Establishing the paleoecology of the turtles from the platy limestones has proven to be taxing, because many specimens are poorly preserved or incomplete and because more complete ones typically lack characters that might uniquely diagnose their habitat preferences. It is therefore not surprising that early work regularly made comparisons with modern pond, river, and marine turtles [6–8], but did not draw firm conclusions regarding their paleoecology. Although explicit comparison with extant turtles remains invaluable when assessing the paleoecology of fossils, a number of recent morphometric studies have raised cautionary notes regarding a straightforward ecological interpretation of turtle anatomy due to unresolved form-function relationships or the lack of clear correspondence of anatomies with a single ecology. For instance, the overall shape of the cranium of extant turtles correlates tightly with marine or terrestrial habitats, a look at the fossil record, however, reveals that turtles with vastly different shapes plausibly occupied these niches as well [9]. Conversely, while a small number of extant turtles show specialized marine versus terrestrial shell shapes, the vast majority of extant turtles possess non-specialized shells that cannot be used to diagnose habitat [10]. Finally, although the relative length of the hand relative to the rest of the forelimb is a good proxy for aquatic adaptations [11], particularly elongate hands are found both in extant marine and freshwater adapted turtles. So, while the shape of skull and shell and the relative length of the forelimbs are telling, accessory data is needed to draw forceful conclusions. Along these lines, the fossil turtle *Eurysternum wagleri* was recently argued to have been an inhabitant of reefs, not only because it appears to have the specialized heart-shaped shell typical of modern marine turtles, but also because it is one of the most common turtles to be found in the platy limestones of Germany, because it is numerous even in the basins more distant from land, because it is known from a range of ontogenetic stages, and, finally, because specimens are known with fossilized gut contents consisting of marine invertebrates, particularly echinoderms [12]. Similar assessments, however, are still lacking for the majority of coeval turtles.

We here describe two unusual specimens from the Late Jurassic platy limestones of southern Germany that provide particularly telling information regarding their paleoecology. The first specimen is a partial hind limb with unusually well-preserved remains of skin, which reveals the development of a broad flipper stiffened by enlarged, well-keratinized scales. Although the specimen lacks taxonomically diagnostic traits for the moment, its attribution to *Thalassemys* sp. appears likely based on associated shell remains. The second specimen is an enormous, articulated skeleton referable to *Thalassemys bruntrutana*, which documents the presence of particularly elongate forelimbs in this Late Jurassic turtle. In concert, the two specimens suggest that at least some turtles from the Late Jurassic of Europe possessed highly keratinized and partially stiffened flippers that are structurally similar to those of extant marine chelonioid turtles. These flippers can best be interpreted as an adaptation to swimming in the open sea.

### Institutional abbreviations

BSPG, Bayerische Staatssammlung für Paläontologie und Geologie, Munich, Germany; CAS, California Academy of Sciences, San Francisco, USA; IRSNB, Koninklijk Belgisch Instituut voor Natuurwetenschappen, Brussels, Belgium; IVPP, Institute of Vertebrate Paleontology and Paleoanthropology, Beijing, China; JME, Jura Museum, Eichstätt, Germany; MB, Museum für Naturkunde Berlin, Berlin, Germany; MJSN, JURASSICA Museum, Porrentruy, Switzerland; MSNM; Museo Civico di Storia Naturale di Milano, Milan, Italy; NHMUK, Natural History Museum, London, United Kingdom; NKMB, Naturkunde-Museum Bamberg, Bamberg, Germany; QM, Queensland Museum, Brisbane, Australia.

### Nomenclature

All names above the species level used herein are formally defined clade names [13] that are highlighted as such through the use of italics.

## Materials and methods

### Geological settings of new material

**Fig 1:**
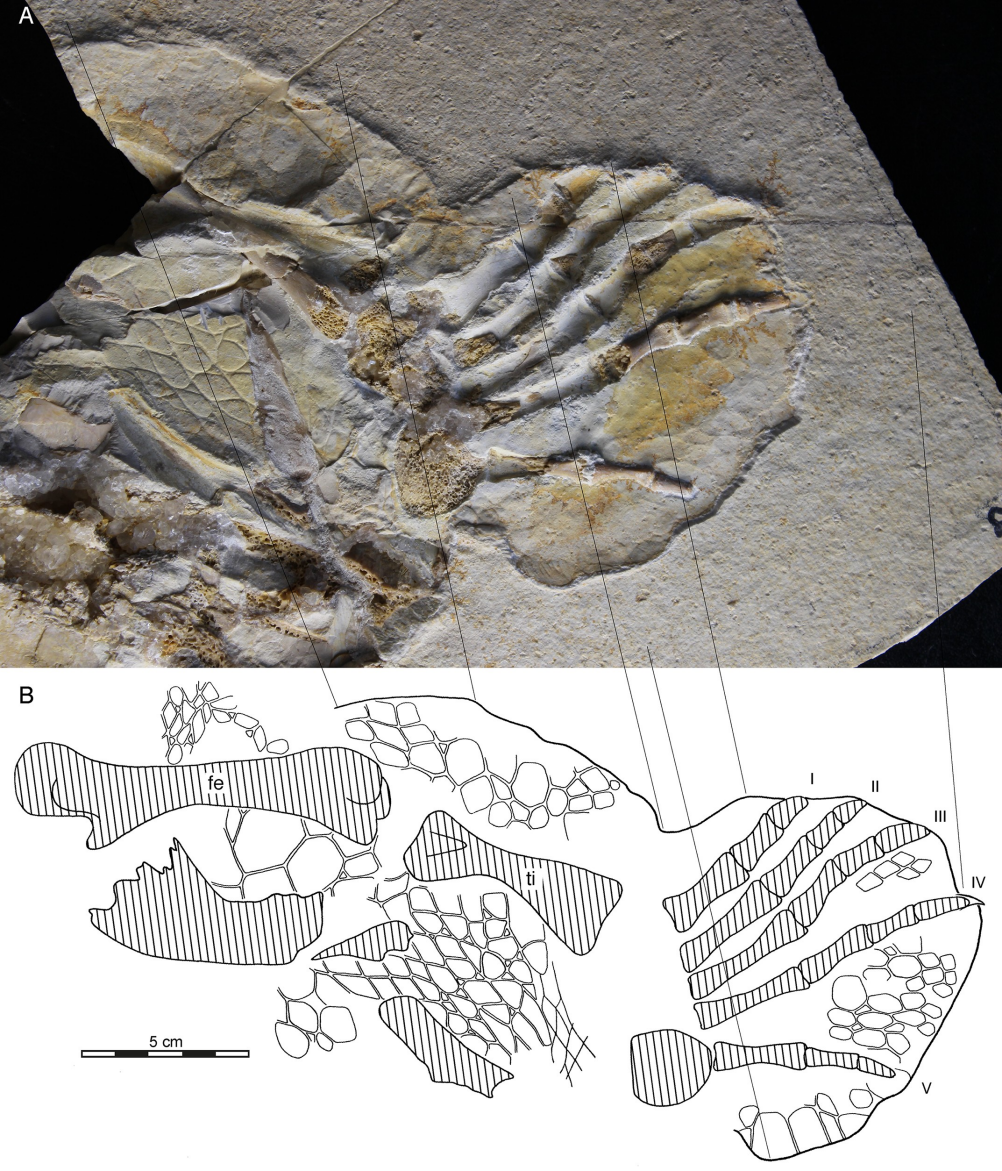
JME 3995, *Thalassochelydia* indet., Late Jurassic (early Tithonian) of Birkhof, Bavaria, Germany. Photograph (top) and interpretive line drawing (bottom) in ventral view. Hatched areas denote bones. Roman numerals denote digit identity. Note large patches of scaly skin preserved across the specimen. For additional images, please see S1 Appendix. Abbreviations: fe, femur; ti, tibia.

Two new fossil turtles are described herein. The first, JME 3995 (Fig 1, S1 Appendix), was collected from a quarry in Birkhof, which is located between Schernfeld and Eichstätt, Bavaria, Germany. The quarry is geologically located within the Blumenberg portion of the Eichstätt basin. The clean micritic matrix of the slab, coloration, and cleavage pattern is typical of the locally quarried lithographic limestones of the Altmühltal Formation (= Solnhofen Formation). These layers have been dated within the Eichstätt basin to the early Tithonian, Late Jurassic [14]. The Altmühltal Formation has yielded a rich assemblage of fossil turtles, including *Eurysternum wagleri* Meyer, 1839a [15], *Idiochelys fitzingeri* Meyer, 1839b [16], *Parachelys eichstättensis* Meyer, 1864 [17], and *Solnhofia parsonsi* Gaffney, 1975 [18]. Although JME 3995 had already been obtained by the JME in 1955, it has not yet been mentioned in the literature, likely because of its incomplete nature. We here tentatively identify it as *Thalassochelydia* indet., while noting important similarities with *Thalassemys* (see Systematic Paleontology below).

**Fig 2:**
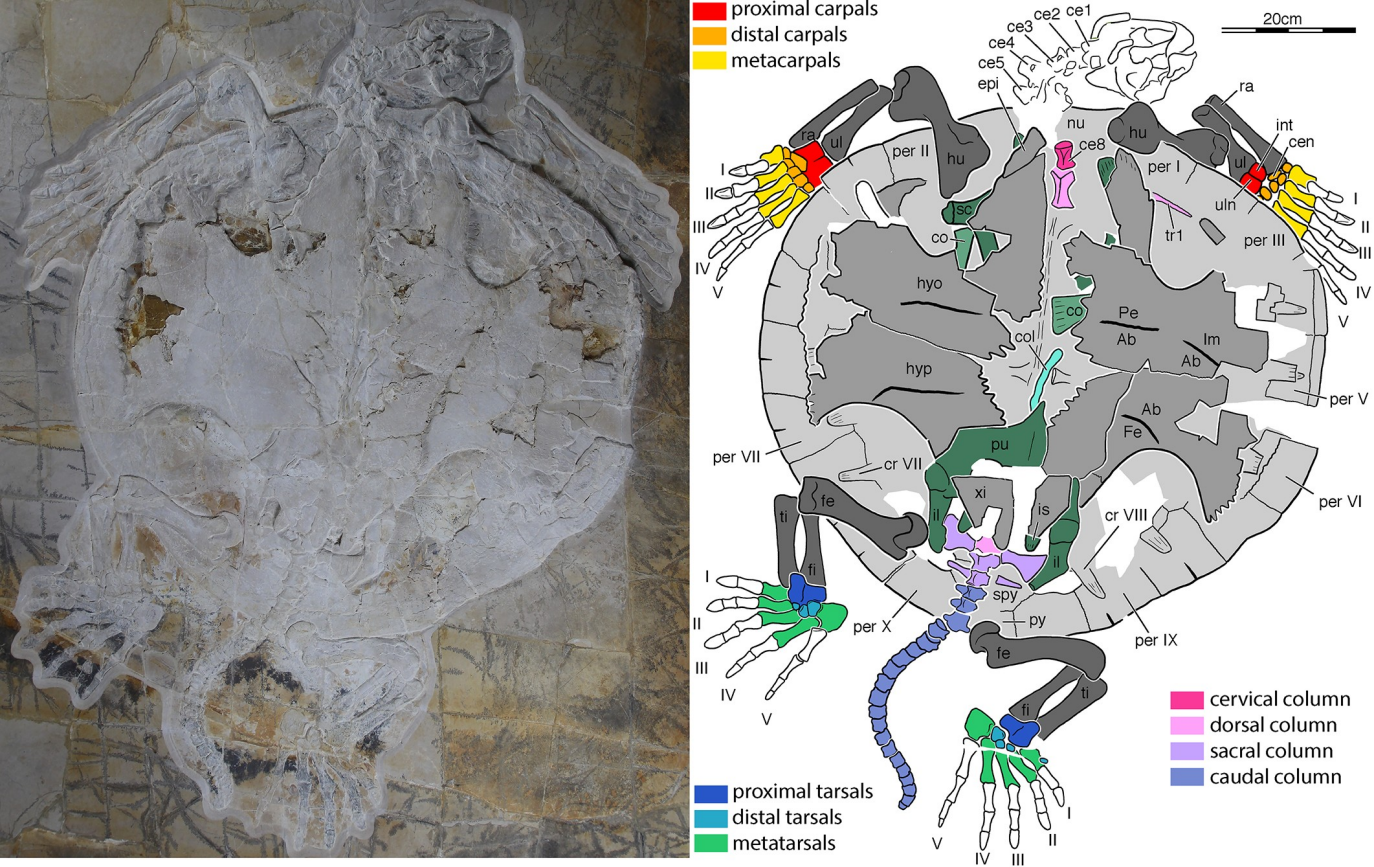
NKMB Watt18/211, *Thalassemys bruntrutana*, Late Jurassic (early Late Kimmeridgian) of Wattendorf, Bavaria, Germany. Photograph (left) and interpretive line drawing (right) of specimen in ventral view. Roman numerals denote digit identity, Arabic numerals vertebral identity. Abbreviations: Ab, abdominal scute; ce, cervical vertebra; cen, centrale; co, coracoid; col, cololite (cololith); cr, costal rib; epi, epiplastron; Fe, femoral scute; fe, femur; fi, fibula; hu, humerus; hyo, hyoplastron; hyp, hypoplastron; il, ilium; Im, inframarginal scute; int, intermedium; is, ischium; nu, nuchal; Pe, pectoral scute; per, peripheral; pu, pubis; py, pygal; ra, radius; sc, scapula; spy, suprapygal; ti, tibia; tr, thoracic rib; ul, ulna; uln, ulnare; xi, xiphiplastron.

**Fig 3:**
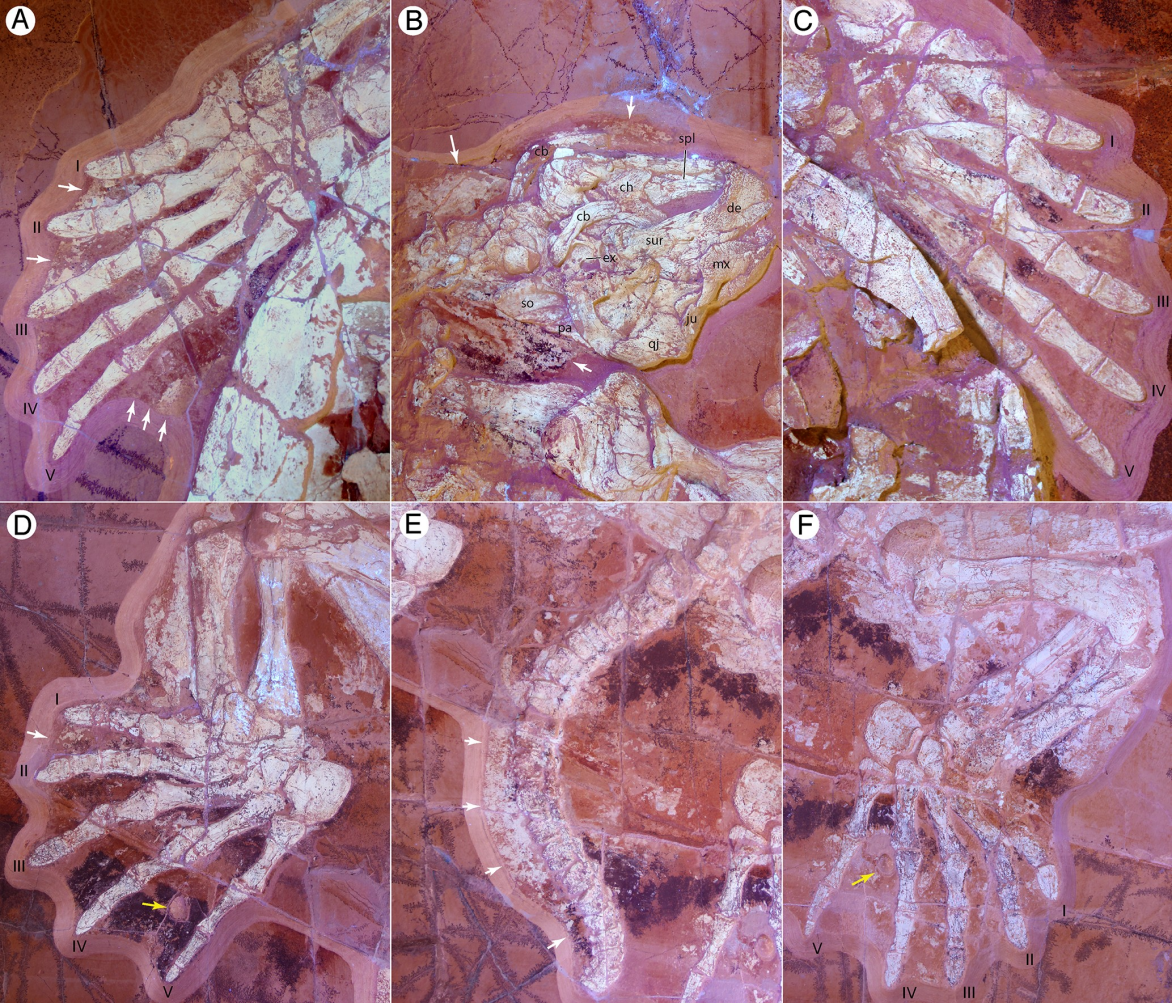
NKMB Watt18/211, *Thalassemys bruntrutana*, Late Jurassic (late Kimmeridgian) of Wattendorf, Bavaria, Germany. Photographs taken under UV light. (A) right forelimb, (B) skull and neck, (C) left forelimb, (D) right hind limb, (E) tail, and (G) left hind limb in ventral view. White arrows highlight soft tissue. Yellow arrows indicate epibionts. For anatomical interpretation of vertebral column and limb, please refer to Fig 2. Abbreviations: cb, ceratobranchial; ch, ceratohyal; de, dentary; ex, exoccipital; ju, jugal; mx, maxilla; pa, parietal; qj, quadratojugal; so, supraoccipital; sur, surangular.

The second specimen, NKMB Watt18/211, is a near-complete skeleton of the thalassochelydian *Thalassemys bruntrutana* Püntener et al., 2015 [19] (Figs 2 and 3). This fossil was collected from the limestone/dolomite quarry of the Andreas Schorr GmbH & Co. KG near the village of Wattendorf in Bavaria, Germany. The fossil originated from a fossiliferous package of platy limestone of only 15 cm thickness as the uppermost part of a sequence of alternating platy limestones with thick layers of reef-debris and graded turbidites, sedimented within a small basin surrounded by dead sponge reefs that has been dated to the base of the early Late Kimmeridgian [20–22]. Systematic excavations of this layer by the NKMB for the last 17 years have yielded an astounding diversity of animal and plant remains [23,24], including exceptionally preserved remains of the thalassochelydian turtles *Achelonia formosa* Meyer, 1860 [6], *Eurysternum wagleri*, *Tropidemys seebachi* Portis, 1878 [25,26]. The absence of bioturbation and ichnofauna in conjunction with remains of benthic invertebrates and articulated vertebrate skeletons suggest that fauna and flora washed into the basin from nearby reefs and landmasses and end up being preserved under anoxic conditions [25]. Ownership of vertebrate fossils is regulated on an individual basis between NKMB and the company owning the quarry. NKMB Wat18/211 is formal property of NKMB and long-term access to scientists is therefore ensured.

### Phylogenetic analysis

We added NKMB Watt18/211 to the phylogeny of Evers et al. (2019) [27]. Although this matrix has its own limitations, we chose it because it samples a relatively large number of potentially marine Jurassic turtles, including sandownids and thalassochelydians, alongside other secondarily marine turtles from the Mesozoic, particularly protostegids and stem-chelonioids. In addition to NKMB Watt18/211, we created a second operational taxonomic unit for *Thalassemys bruntrutana* on the basis of scorings for NKMB Watt18/211 plus recently described and illustrated specimens from Switzerland [19] and the United Kingdom [28], which provide additional information for characters pertaining to the morphology of the carapace.

We modified the definition of character 348 [27] to encode whether the fourth manual digit is longer than the third (state 0) or the third is longer than the fourth (state 1). The original character definition coded whether the fourth or third manual digits are the longest in the manus, and was intended to capture variation in hand architecture between protostegids and *Toxochelys* sp. on one side, in which the fourth digit is longer than the third [27,29], and crown chelonioids on the other side, in which the third digit is the longest of the manus [27]. NKMB Wat18/211 has a morphology in which the fifth digit is the longest of the manus, thus showing previously uncoded variation. This morphology was not applicable under the previous character definition. Instead of adding a state (“fifth digit is longest in manus”), we now relate the length of the fourth digit to the length of the third. Following this definition, NKMB Watt18/211 can be scored the same as protostegids, reflecting that these taxa share an elongation of the fourth finger. We also added a new character (character 356) that differentiates between the plesiomorphic orientation of zeugopodium of the forelimb, where, depending on the step cycle, the radius is located medial or posterior to the radius, and its derived orientation, where the radius is located anterior to the ulna due to the permanent hyperextension of the elbow joint.

In addition, we modified two character states for *Solnhofia parsonsi* and *Plesiochelys etalloni* Pictet and Humbert, 1857 [30] (each from 216.1 to 216.0, specifying the absence of rib-free peripherals), which had previously been typographically mis-scored [27]. The full list of character is provided in S2 Appendix the character/taxon matrix in S3 Appendix.

We analyzed the dataset using TNT 1.5 [31,32] with previous settings [27]. In particular, *Proganochelys quenstedtii* was set as the outgroup, a backbone constraint for the topology of extant taxa was enforced [33], and all characters were treated as equally weighted and unordered. We used the new technology search with the default setting, tree drifting, and parsimony ratchet enabled [34,35], an initial level of driven search of 30, with the number of times the minimum tree length to be obtained as 30. The most parsimonious trees (MPTs) of this initial run were subjected to a further round of tree bisection and reconnection (TBR).

## Systematic paleontology

*TESTUDINATA* Klein, 1760 [36]

*THALASSOCHELYDIA* Anquetin et al., 2017 [1]

*Thalassochelydia* indet.

Fig 1

### Referred specimen

JME 3995, an articulated left hindlimb associated with the anterolateral portions of the carapace (Fig 1) from Late Jurassic (early Tithonian) Altmühltal Formation of Birkhof, Bavaria, Germany.

### Comments

See the Description below for the morphology of JME 3995 and the Discussion for its referral to *Thalassochelydia indet*.

*Thalassemys* Rütimeyer, 1873 [8]

### Type species

*Thalassemys hugii* Rütimeyer, 1873 [8].

*Thalassemys bruntrutana* Püntener et al. 2015 [19]

Figs 2 and 3

### Type specimen

**Fig 4:**
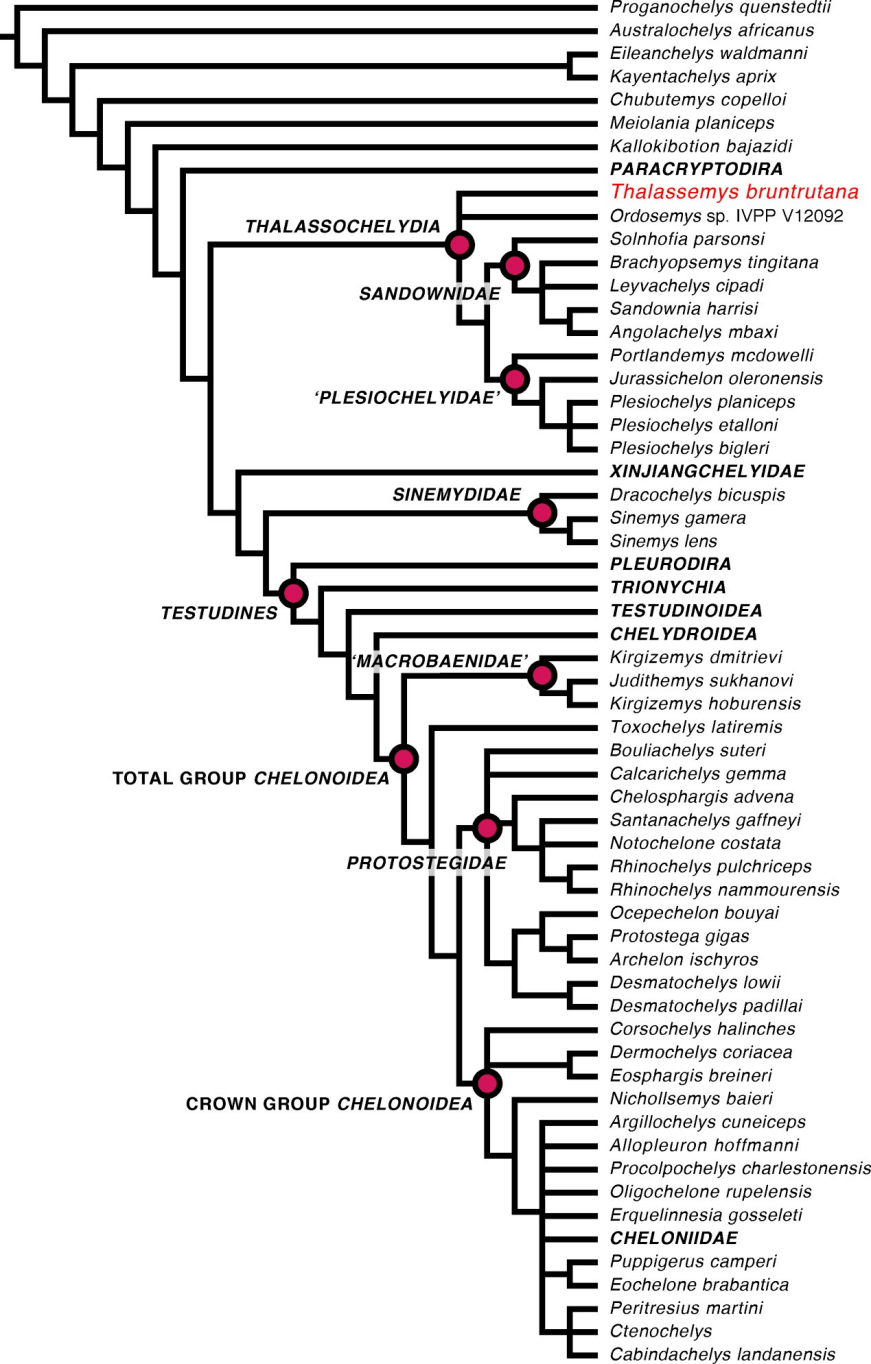
Strict consensus of 5940 MPTs of 1743 steps showing the phylogenetic placement of *Thalassemys bruntrutana* as well as important clades and taxa discussed in the text. Note that terminal clades in bold were been collapsed to save space (see S4 Appendix for full topology). Important internal nodes are labelled. ‘Plesiochelydidae’ is placed in single quotes to indicate that is does not have a formal phylogenetic definition. ‘Macrobaenidae’ is also placed in single quotes because the specifier for this clade, *Macrobaena mongolica*, is not included. However, the respective taxa are commonly labelled as ‘macrobaenids’ in the literature.

**Fig 5:**
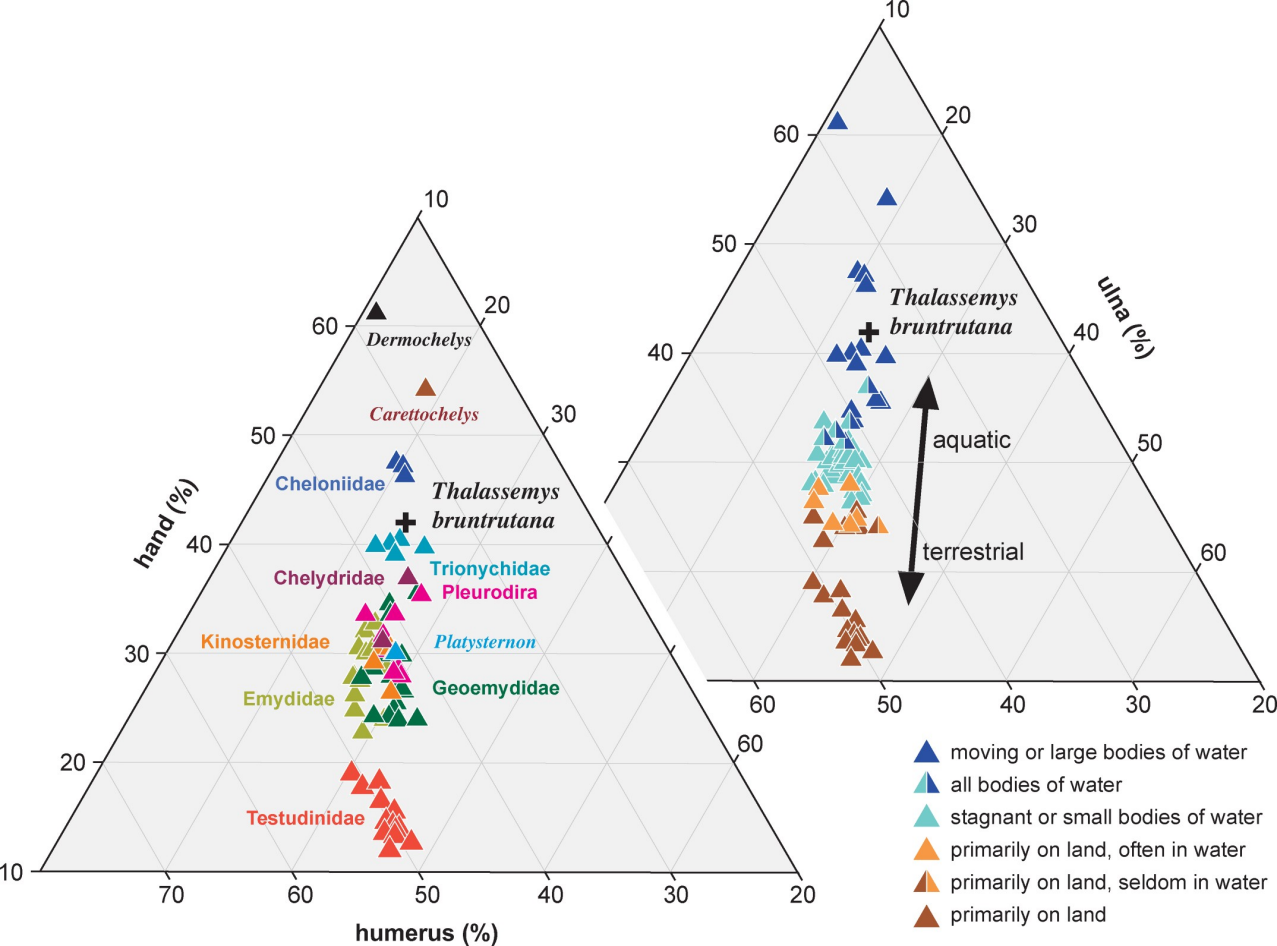
The relative length of the humerus, ulna, and hand of *Thalassemys bruntrutana* relative to that of extant turtles. In the diagram to the front, extant turtles are color coded for taxonomic groups, in the diagram to the back, extant turtles are color coded for habitat preferences. All data of extant turtles are taken from Joyce and Gauthier (2004) [11]. Note that *Thalassemys bruntrutana* has relatively longer hand that extant trionychids, but shorter hands that extant carettochelyids and chelonioids.

**Fig 6:**
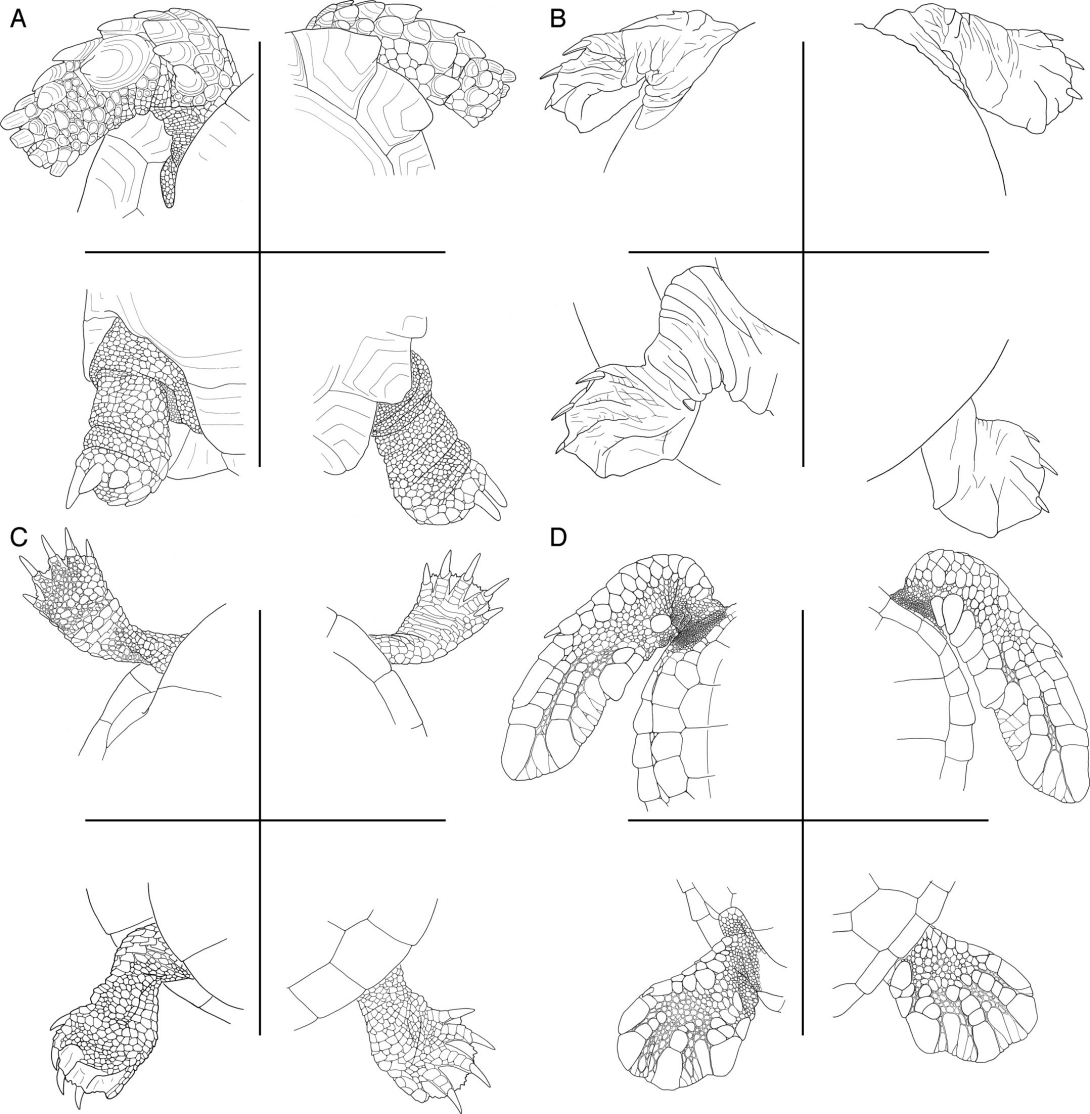
The comparative anatomy of the skin of the limbs of extant turtles. (A) the testudinid tortoise CAS 165598 *Psammobates oculifera*, an inhabitant of desserts. (B) the trionychid turtle CAS 65705 *Apalone spinifera*, an inhabitant of rivers. (C) the emydid turtle CAS 13889 *Chrysemys picta*, an inhabitant of ponds. (D) the cheloniid turtle CAS 8478 *Chelonia mydas*, an inhabitant of oceans.

MJSN SCR011-87 (holotype), a partial shell (Püntener et al. 2015 [19], Figs 3–6).

### Type locality and horizon

Courtedoux, Canton of Jura, Switzerland; Lower Virgula Marls, Chevenez Member, Reuchenette Formation, late Kimmeridgian, Late Jurassic [19].

### Referred specimen

NKMB Watt18/211, a flattened, nearly complete skeleton (Fig 2) from the Late Jurassic (early Late Kimmeridgian) of Wattendorf, Bavaria, Germany.

### Distribution

Late Jurassic (late Kimmeridgian) of Courtedoux, Switzerland (including the holotype [19]); Egmont Bight, Isle of Purbeck, United Kingdom [19,28], and Wattendorf, Bavaria, Germany (specimen referred herein).

### Emended diagnosis

*Thalassemys bruntrutana* can be diagnosed as a member of *Thalassochelydia* by the likely presence of three cervical scutes and as a member *Thalassemys* by large size (carapace length greater than 60 cm); development of a round carapace that lacks a distinct nuchal notch; absence of costo-peripheral fontanelles in adults; vertebral scutes with anterolaterally concave and posterolaterally convex margins; presence of an osseous bridge in adults; presence of lateral and central plastral fontanelles in adults; absence of sutural contact between the hyoplastra and the more anterior plastral elements; and a scapular angle greater than 110°. *Thalassemys bruntrutana* differs from *Thalassemys hugii* by the presence of a more elongated nuchal with strong anterolateral thickening on the ventral surface; broader vertebral scutes; a proportionally longer, but broader plastron with triangular lobes and less inclined xiphiplastra; presence of separate lateral, central, and xiphiplastral fontanelles; and a wider scapular angle. *Thalassemys bruntrutana* differs from *Thalassemys marine* by the development of broader vertebral scutes. *Thalassemys bruntrutana* further differs from other thalassochelydians by the presence of an elongate cranium and mandible with moderate lower temporal emargination and an elongate symphysis; 10 pairs of peripherals; strap-like epiplastra that bluntly cover the anterolateral margins of the hyoplastra; and manus and pes that are transformed into elongate stiffened paddles, but it is unclear how these characters are developed within *Thalassemys* (modified from [1,19]).

### Comments

See the Description below for the morphology of NKMB Wat18/211 and the Discussion for its referral to *Thalassemys bruntrutana*.

## Descriptions

### JME 3995 –*Thalassochelydia* indet

JME 3995 consists of part of a shell and an associated left hindlimb preserved on two slabs (Fig 1, S1 Appendix). The overall preservation suggests a predation event during which a turtle was crushed, but only partially consumed. The articulated portion of the shell (see S1 Appendix) only includes the lateral aspects of left costals I and II, parts of peripherals II–V, and the anterior tip of the axillary buttress. The narrow peripherals and the long free rib ends of the costals frame extensive carapacial fontanelles. A series of unidentified peripherals are located behind the limb. Additional fragments are strewn across the specimen. The hind limb likely came to rest intact as all bones show good 3D preservation, although most elements were damaged during the splitting of the slab. The pes resembles most other known turtles from the platy limestones of Europe by having an enlarged hooked fifth metatarsal (= ansula) and a digital formula of 2-3-3-3-3.

The most notable feature of JME 3995 is the exquisitely preserved skin of the hind limb. The soft tissue manifests itself in part in the form of a phosphatic discoloration that rises above the surrounding sediment. Such soft-tissue "shadows" are commonly preserved among the turtles of the platy limestones of Europe (pers. observations of published specimens), but rarely described in detail, as morphological details are lacking. In contrast to most previously documented specimens, scales can be made out across both slabs, especially under low light (Fig 1A, S1 Appendix). The scales are boldly impressed in some areas, but only faint in others. We are not able to clarify if the scales document the dorsal or ventral side of the limb. Our illustration (Fig 1B) therefore perhaps represents a composite of both sides. Just above the femur, a small patch of skin is apparent consisting of numerous small scales that are organized transverse to the direction of the femur. The resulting folds in the skin may have accommodated mobility at the base of the leg. For the remainder of the upper and lower thigh, large patches of skin are visible consisting of medium to large-sized scales that lack a clear orientation. Just below the lower thigh, smaller scales are apparent that are oriented in rows perpendicular to the tibia, which likely supported the movement of the ankle. The outline of a flipper is preserved as a raised pedestal of phosphate that surrounds the foot. Patches of scales are apparent between digits III, IV, and V, including a scale that surrounds the claw of digit V. In addition, a flap of skin is developed along the trailing edge of the flipper that is stiffened by medium sizes scales along its margins.

### NKMB Watt 18/211 –*Thalassemys bruntrutana*

NKMB Watt18/211 is a visually stunning specimen exposed in ventral view, but many morphological details are obscured by taphonomic crushing and tectonic fracturing, which impede the collecting process (Figs 2 and 3). Although difficult to assess from the articulated specimen, we estimate that the full skeleton was flattened to a thickness of about 1cm. This collapse was associated with fracturing and displacement, which likely took place early in diagenesis, followed by plastic deformation, particularly to the long bones, vertebrae, and skull. The specimen furthermore suffered much damage while collecting, as the platy limestones at Wattendorf in some areas do not split into large slabs, but rather in hundreds of polygonal elements that must be pieced together again in the lab. Some bone fragments were lost in the process. The low color contrast between the matrix and the bone can partially be compensated through the use of UV light (Fig 3). Many morphological details, nevertheless, remain unclear. As preserved, the carapace has a length of 82 cm and a width of 85 cm. It is therefore marginally smaller than a partial, coeval specimen from England referred to *Thalassemys hugii*, which is estimated to have reached about 95 cm in carapace length [37]. A summary of other basic measurements is provided in Table 1.

**Table 1:** A selection of basic measurement of NKMB Watt18/211.

Element	Measurement	Value [cm]
Cranium	Length (premaxilla to basioccipital)	14.0
Carapace	Length	82.0
	Width	85.0
Right forearm		
Humerus	Length	19.0
Radius	Length	16.2
Ulna	Length	13.7
Manus	Length (proximal carpals to tip of digit I)	26.0
Digit I	Length (including metacarpal)	18.7
Metacarpal I	Length	6.7
Digit I phalanx 1	Length	5.2
Digit I phalanx 2	Length	4.1
Digit I phalanx 3	Length	2.7
Right hindleg		
Femur	Length	20.0
Tibia	Length	14.2
Fibula	Length	NA
Metatarsal IV	Length	7.0
Digit IV phalanx 1	Length	4.2
Digit IV phalanx 2	Length	3.0
Digit IV phalanx 3	Length	3.0

#### Cranium

Although the cranium of NKMB Watt 18/211 is preserved in its entirety, we are only able to glean minimal details from the available ventrolateral view due to significant fragmentation, displacement, and crushing (Figs 2 and 3B). The cranium at first sign looks to be rather broad, but the well-preserved mandible suggests that it was elongate. The outer surface of the short but high left maxilla is thickly textured. As the specimen otherwise exhibits unambiguous soft-tissue preservation, we speculate that the phosphatized rhamphotheca may be preserved. A moderate lower temporal emargination is developed posterior to the maxilla. The left jugal is exposed in this area in a relatively high position dorsally above the labial ridge of the maxilla. Posterior to the jugal, the left quadratojugal can be seen, with a rounded posterior margin that likely framed the anterior margin of the cavum tympani, a broad posterodorsal process that likely arched over the cavum tympani, but only a small, or a damaged posteroventral process. In all regards, this part of the cranium corresponds to that of *Jurassichelon oleronensis* [38]. A series of elements are preserved between the left temporal arch and the mandible, but we are unable to identify these with confidence, although we speculate that the largest represents the parietal in ventral view. At the posterior end of the cranium, the supraoccipital crest is apparent. The length of the supraoccipital crest suggests that it protruded significantly beyond the level of occipital condyle. A small piece of bone is apparent above the supraoccipital that we interpret as the posterior tip of the parietal. A small bone with two small foramina is exposed near the ventral base of the supraoccipital crest that likely represents the left exoccipital and hypoglossal nerve foramina.

#### Mandible

The mandible of NKMB Watt 18/211 is well exposed in ventral view, but crushing and damage once again obscured many details (Figs 2 and 3B). The mandible is elongate and has a well-developed, anteriorly sloped symphysis about 30% of the length of the mandible, although significant distortion makes it difficult to take objective measurements. The external surface of the anterior third of the mandible is thickly textured, which we speculate may represent the phosphatized rhamphotheca. A broad and high coronoid process rises above the triturating surface, but is partially obscured by disarticulated cranial bones. The dentary-surangular suture is clearly visible behind the coronoid process. A narrow bone is apparent on the inner side of the right ramus that we interpret as the splenial.

#### Hyoid apparatus

Three bones are preserved below the mandible of NKMB Watt18/211 that we interpret as the unpaired ceratohyal and the paired ceratobranchial bones (Figs 2 and 3B). The ceratobranchials are boomerang-shaped, compact rods that retain approximately the same thickness for their entire length. The ceratohyal is a plate-like element that is mediolaterally broader than anteroposteriorly long and centrally constricted between the posterior and anterior lateral processes. As preserved, the anterolateral processes are longer than the posterolateral processes and form lobe-like projections that diverge slightly from either side of the midline.

#### Carapace

As preserved, the carapace of NKMB Watt18/211 is mostly rounded, but there is a hint of the tear-drop shape typical of extant marine turtles, as the nuchal and pygal regions protrudes slightly. The carapace appears to lack fontanelles, with exception of a small window developed between costal VIII, the suprapygals, and peripheral X. The rib ends of costal ribs III to VIII insert superficially in the middle of the underside of peripherals IV–IX. We are only able to count 10 pairs of peripherals, but the nuchal region is difficult to interpret, as the limbs and neck cover the region. It is possible that NKMB Watt18/211 possess eleven pairs of peripherals, but the available space would only allow for a narrow nuchal and narrow anterior peripherals. However, as *Thalassemys* species are otherwise known to have broad nuchals and broad anterior peripherals [19,39], we find it more likely that only 10 pairs of peripherals are present. The bridge reaches from the middle of peripheral II to the middle of peripheral VII. The rectangular pygal is visible below the tail, which is slightly narrower, but much shorter than the surrounding peripherals. At least two suprapygal elements are present anterior to the pygal, which contribute to the abovementioned carapacial fontanelle. The poorly impressed sulci of the marginals are only apparent near the outer rim of all peripheral elements.

#### Plastron

Although the shell is preserved in ventral view, the plastron is difficult to interpret in parts, as all elements slightly disarticulated and shifted during taphonomy and because much bony material was lost during recovery. We identify an elongate bone along the anterior tip of the plastron as the right epiplastron. This triangular element likely contacted its counterpart along the midline, but otherwise bluntly abuts against the anterolateral margins of the hyoplastra. There is no trace of an entoplastron. The remainder of the plastron consists of paired hyo-, hypo-, and xiphiplastra. The straight medial margin of the right hyoplastron and xiphiplastron suggests that these elements contacted their counterparts along the midline. The hyo- and hypoplastra otherwise frame an irregular central plastral fontanelle. The lateral aspect of the hyo-hypoplastral contact is damaged on both sides, but the wavy medial margins of the sixth and seventh peripherals suggest that lateral plastral fontanelles are absent. We are unable to find the conspicuous striation that run perpendicular to the hyo-hypoplastral suture in the holotype of the species [19]. The hyoplastra form elongate axillary processes that terminate near the center of peripheral II. It is unclear if a costal contact is present. The inguinal buttress of the right hypoplastron, preserved in articulation on the right side, terminates at the middle of peripheral VII. A costal contact is clearly absent. The bridge itself is formed by numerous pegs of the plastron that insert into sockets formed by the carapace. Around the perimeters of the central plastral fontanelles, portions of the pectoral-abdominal and the abdominal-femoral sulcus are apparent. The humeral-abdominal sulcus is located far behind the level of the axillary notches and oriented transversely. The abdominal-femoral sulcus is mostly oriented transversely as well, but the lateral aspects bend posteriorly to end within the inguinal notch. A short, diagonally oriented sulcus is furthermore visible on the left hyoplastron, which we interpret as the sulcus between an inframarginal and the abdominal.

#### Cervical vertebrae and ribs

The cervical series of NKMB Watt18/211 seems to be complete, but overall preservation within the neck region is poor (Figs 2 and 3B). Cervicals 1–5 are exposed in left lateral view and cervical 8 in ventral view. The identity of the remaining cervicals is unclear. From what can be seen, the anterior cervical vertebrae have robust prezygapophyses, best seen in cervical 3 and cervical 4 (Fig 3B). Neural spines appear to be absent or very low. The anterior centra are unkeeled, and the preserved transverse processes are thick, round in lateral outline, and posteriorly removed from the anterior central margin so that they assume a nearly central position along the anteroposterior length of each vertebra, most apparent in cervical III and IV. Cervical VIII has a low but robust ventral keel, which spans nearly across the entire length of the centrum (Fig 2). Anteriorly, the keel merges with a transverse ridge on the underside of the centrum. The centrum of cervical VIII is anteriorly broadened with respect to the posterior parts of the same vertebra, but a double articulation is seemingly absent. Instead, the anterior surface of the cervical VIII is vaguely concave. The full neck is likely no more than 30 percent of the length of the carapace. NKMB Watt18/211 is therefore notably short-necked [40].

#### Dorsal vertebrae and ribs

The first two dorsal vertebrae are exposed in ventral view between the hyoplastra, which are disarticulated along the midline (Fig 2). The first dorsal vertebra has a concave anterior articulation facet that appears to be gently downturned ventrally. The vertebral bodies of these vertebrae are hour-glass shaped between its articular ends. A shallow but distinct keel is visible on the ventral surface of the first, but not the second dorsal vertebra. A thin ridge traverses the underside of the carapace in approximate continuation of the more anterior dorsal vertebrae. Although we cannot detect vertebral segments, we carefully interpret this as the strongly altered remainders of the dorsal column. The distal end of the first thoracic rib is visible within the left axillary notch. Although the margins of the costal it underlies cannot be traced, it is apparent that first thoracic rib likely spanned much of the width of the costal.

#### Sacral vertebrae and ribs

The first sacral vertebra is fully exposed posterior to the xiphiplastra and in articulation with the posterior end of the last dorsal vertebra in NKMB Watt18/211 (Fig 2). The vertebral centrum of the sacral I is slightly more robust and longer than the following vertebrae, but shorter than the dorsal vertebrae. Anteriorly, sacral I has strong and robust transverse processes that project sideways like wings and connect to the first sacral rib. The first sacral rib is blade-like and centrally constricted between its articulation surfaces with sacral I and the ilium. The ilial side of the first sacral rib is expanded to approximately twice the width of the proximal end that faces the sacral vertebra. The second sacral vertebra and ribs are smaller than the first, particularly the rib. Although some caution should be applied with interpreting the contacts due to breakage along the sacral ribs and the posterior tip of the left ilium, it seems that the second sacral rib was not in direct contact with the ilium and that there was certainly no strong connection between the two, if at all. This is best seen on the right side of the specimen, where the ilium is complete and in full articulation with the first sacral rib. Although the second sacral rib is complete and in articulation with the second sacral vertebra, there is about a centimeter space between the posterior part of the ilium and the distal end of the right second sacral rib. The right second sacral rib is rounded at its tip and shows no indication of breakage. The left side only partially confirms these observations as the posterior tip of the ilium is slightly damaged, and the second sacral rib is broken into two pieces. However, the preserved region gives no indication to believe that there was a contact between the ilium and the second sacral rib. Thus, we interpret the second sacral vertebra to be freed from any functional integration into the sacrum. As turtles usually have two sacral vertebrae, we retain this vertebra nomenclaturally as a ‘sacral vertebra’ here, but note that it is caudalized.

#### Caudal vertebrae and ribs

Posterior to the second sacral vertebra, 21 true caudal vertebrae are preserved in NKMB Watt18/211 (Figs 2 and 3E). It seems that the tail is complete, as the last caudal is a small knob of bone with a blunt posterior surface that does not seem to allow for the articulation of further elements. The caudal vertebrae become successively smaller posteriorly. The nature of their articular surfaces is not entirely clear. Vaguely triangular notches are conspicuous on the ventral side that are formed between two adjacent caudal vertebrae and that can be observed in several pairs of caudals. There is no unambiguous evidence for the presence of chevrons, but the negative intercentral topology could justify the inference of small chevrons, although it is unclear why none of them should have been preserved given the articulated state of the fossil. Although the pelvis and sacrum may have shifted posteriorly when the carcass partially disarticulated early in taphonomy, the tail clearly must have protruded beyond the posterior margin of the shell. In concert with the robust morphology of the caudals, this suggests strongly that the individual present is a male [26,41].

#### Pectoral girdle

Most of the pectoral girdles are underlain by the plastron of NKMB Watt18/211, but breakage in the latter exposes parts of both (Fig 2). For instance, between the nuchal and right epiplastron, there is a short exposure of the scapular (= dorsal) process of the right scapula. The same element can be traced further posteriorly, between the hyoplastron and proximal humerus end, where the glenoid region of the right scapula is exposed. This region shows a clear and relatively long glenoid neck, similar to the condition in total-group chelonioids. Posteriorly to the glenoid neck, a short part of the acromion process is exposed. The angle between the processes of the scapula can be estimated to have been relatively wide and certainly larger than 90°. The ventral ridge of the scapula between the acromion process and glenoid is likely absent, but we cannot be absolutely certain of this as the scapula is not perfectly oriented to assess this feature. However, the horizontal ridge of the scapula which forms a web of bone between the glenoid end and the coracoid is likely absent, as there is no indication of such a bony web at the proximal coracoid end that is preserved just ventral to the acromion process of the right scapula. The glenoid end of the scapula is 26 mm wide across its widest point. Parts of the left scapula are exposed as well, in particular the dorsal end of the scapular process, which shows distinct striations. On the left side of the specimen, the distal end of the left coracoid is exposed near the midline of the skeleton, within the central fontanelle, while the portion of the left coracoid is exposed posterior to the left glenoid. Although the full length of the coracoid is not documented, these two fragments suggest that the coracoid is at least twice as broad distally than near its glenoid end.

#### Forelimbs

The forelimbs are almost completely preserved in NKMB Watt18/211 (Figs 2, 3A and 3C). On the left side, the humerus is disarticulated from the antebrachium, but the forearm itself and the hand are in articulation. The entire right arm is fully articulated. Both humeri are plastically deformed around the anterior rim of the carapace, as their proximal ends lie within the axillary opening between plastron and carapace. The humeri are exposed with their ventral sides. The right humerus is better preserved and shows that the proximal head is broad and that a large medial process is present. Along the proximal end of the humerus, the medial process is separated from the humeral head by a shallow notch. The humeral head is largely invisible as it is located on the dorsal surface of the humerus, but its outline suggests that is was well rounded. The lateral process of the humerus is positioned slightly distally to the humeral head, but still near the proximal end of the humerus. Its position is similar to that seen in the stem-chelonioid *Toxochelys* spp. [27]. The distal end of the humerus is as broad as the proximal end. The articulation facets for the antebrachium are developed as a clear bi-condylar surface on the ventral side of the humerus. It is unclear if the ectepicondylar foramen is formed as a true foramen or as a groove, as this feature is expected on the unexposed dorsal surface. The proximal ends of the ulna and radius are aligned on the same level on both sides, indicating that these bones are preserved in their original position. As in most turtles, the proximal end of the ulna is not expanded with regard to its shaft, and an olecranon process is absent. However, in contrast to all continental turtles, the radius is positioned towards the anterior, suggesting that the elbow was permanently hyperextended, as in chelonioids. Distally, the ulna broadens significantly and forms a broad articulation surface for the proximal carpal bones. The radius is narrower than the ulna, but also much longer. It extends laterally along the intermedium and comes close to contacting the distal carpal I. On both hands, there is some space between the distal carpal I and the end of the radius that was likely filled with cartilage in life, possibly representing a cartilaginous radiale, which is generally absent as an ossified element in turtles [42]. The ulnare and intermedium are approximately the same size, and the ulnare is the medial of the two proximal carpals. Both are aligned to one another, but neither fused nor sutured. The medial and lateral centralia are fused to a single, mediolaterally broad, lens-like element that is wedged between the proximal and distal carpals. The pisiform is concealed by the carapace on the right side, but peaks from below the carapace on the left side. We are nevertheless unable to provide meaningful anatomical observations. The five distal carpals are all subequal in size and mediolaterally aligned in a row without any space between them. The distal carpals are also closely articulated with the metacarpals, whereby each distal carpal I–V closely aligns with the positions of the proximal ends of metacarpals I–V. Each metacarpal is slightly overlapped by its medial neighbor along the proximal end of the element. Metacarpal I is relatively short and broad, whereas the other metacarpals are elongated. Metacarpals II, III, and IV become successively longer than the preceding metacarpals, and the fifth metacarpal is as long as the fourth. However, the digits in their entire lengths become successively longer from I–V. This length increase is facilitated by the phalangeal formula 2-2-3-3-3, the shortness of the first metacarpal with respect to the second, and a length increase in the penultimate phalanges of fingers III–V. There is evidence for interphalangeal movability in some digits. In digits I and II, the proximal end of the first phalanges have well-developed flexor tubercles that overlap the distal metacarpal ends. The same morphology, albeit weaker in development, can be seen in digit III. Conversely, in digits IV–V, the phalangeal ends abut one another is a planar surface, which is much more similar to the condition of extant chelonioids which have almost rigid interphalangeal articulations. The first three digits are additionally assessed with relatively robust unguals. It is possible, that the movability of the first three digits in this specimen indicates grasping capabilities as seen in males of extant chelonioids, and thus represents a sex feature [43]. Alternatively, movable first digits and ridged last digits have been proposed as an incomplete adaptation to marine locomotion in stem chelonioids like *Toxochelys* or protostegids [27,44]. The unguals of the first three digits are short but broad. The fourth digit of NKMB Watt18/211 also bears an ungual, but this element is slightly smaller than in the preceding digits. The fifth digit has a miniaturized ungual, although it is not clear if it was truly covered by a keratinous sheath during life. The second phalanx of the fifth digit of the right manus shows a callus-like thickening along its midshaft, suggesting that the finger element suffered from a fracture that was healed.

#### Pelvic girdle

Parts of the pelvic girdle of NKMB Watt18/211 are exposed around the preserved parts of the xiphiplastra, including both ilia, portions of the right pubis, nearly the complete right pubis, and parts of the ischia (Fig 2). On both sides, the ilium is preserved in articulation with the first sacral rib. The posteriormost tip of the left ilium is broken, but comparisons with the right side and the length of the preserved second ‘sacral’ ribs on both sides indicate that only these ribs lack contact with the ilium (see sacrum above). The pubes are exposed in part by breakage of the plastron. Both pubes are dorsoventrally flattened, and their midline contact is visible for a short distance posterior to the central plastral fontanelle. Most of the right pubis is exposed, in ventral view. At the anterior end, the left pubis forms a tapering epipubic process that is broken off in its left counterpart. Anterolaterally, the lateral pubic process is overlain and completely concealed by the right hypoplastron. Posteromedially, the right pubis has a concave margin toward a large thyroid fenestra, but it is unclear if it coalesced with its counterpart. The posterior tips of both metischial processes of the ischia are apparent behind the xiphiplastra.

#### Hindlimbs

Both hindlimbs are preserved completely and in near-perfect articulation in NKMB Watt18/211 (Figs 2, 3E and 3F). The left femur is exposed in posterior view. It is posteriorly displaced from the pelvis with its proximal end, but the distal end is still articulated with the zeugopodium. The complete proximal end of the left radius is hereby overlain by the femur whereas parts of the proximal end of the tibia are exposed. On the right side, the femur is slightly more disarticulated, as the proximal end is removed from the acetabulum and additionally the element is rotated outward around its long axis, exposing the tibial and fibular articulation facets. Thus, the right femur is exposed in anteroventral view, so that all major sides that bear anatomically diagnostic features are exposed between both femora. The femoral head is directed anterodorsally away from the axis of the femoral shaft and is well rounded. The minor trochanter, which is only visible on the right femur, is a relatively prominent process that ends well below (= distal to) the femoral head surface. The major trochanter, visible in both femora, is also a distinct and low process, which is separated from the femoral head surface by a deep notch. This is unlike the condition convergently shared between early stem group turtles like *Proganochelys quenstedtii* [42] and crown-group *Chelonioidea* [27], in which the major trochanter and femoral head form a continuous epiphyseal surface. However, the condition in NKMB Watt18/211 is similar to that seen in most non-chelonioid crown-group turtles, including the stem-chelonioid *Toxochelys* spp. [27]. A shallow intertrochanteric ridge is present in NKMB Watt18/211 between the major and minor trochanter, but the ridge is low and concave along its margin. This ridge is absent in *Toxochelys* spp., but present in most chelonioids [27]. The femoral shaft of NKMB Watt18/211 is relatively thick and only weakly constricted with regard to the width of the proximal and distal ends of the element. Although this is likely hypertrophied by the compression of the fossil, the femora appear to be genuinely robust. The distal end of the femur bears two weak condyles, whereby the tibial condyle is slightly larger than the fibular condyle. Tibiae and fibulae are preserved in their approximate natural positions on both sides of the specimen, with each tibia being laterally adjacent to the fibula. Tibia and fibula are gently constricted along their midshaft regions, creating a lenticular space between both elements. The proximal fibular ends are concealed by the femora on both sides, as is the proximal end of the left tibia. For the right tibia, the proximal end is visible, but somewhat flattened so that no distinct features are discernible. Tibia and fibula are of the same length, and their distal ends are aligned with one another. The fibula is distally broader than the tibia, whereas the opposite is true for the proximal ends of both bones. The astragalus and calcaneum are sutured to one another, but not fused, as a vertical suture line is visible between astragalus and calcaneum on both sides. The calcaneum is broad mediolaterally and short proximodistally and lies entirely below the distal end of the fibula. The astragalus is proximodistally longer than it is mediolaterally wide and extends beyond the level of the calcaneum both proximally and distally. Proximally, the posterior end of the astragalus overlaps both the fibula and tibia, as is usual for turtles [42]. Only three distal tarsals are visible in the right ankle, but the expected four distal tarsals are clearly visible on the left side. The fourth distal tarsal is the largest. It has a proximal surface that articulates relatively tightly with the calcaneum. Medially, the fourth distal tarsal forms a straight medial margin, toward which the hooked fifth metatarsal (= ansula) abuts. Distally, this margin curves gently laterally and forms a short surface toward the fourth metacarpal. Laterodistally, the fourth distal tarsal has a sinuous margin along which it tightly articulates with the third distal tarsal. The latter element is smaller than the fourth distal tarsal and concavely rounded along the sides that face the second distal tarsal and the metatarsals. The second and first distal tarsals become successively smaller and are positioned laterally to the third distal tarsal and between the astragalus and metatarsals. The first through fourth metatarsals become successively longer. The first metatarsal is more robust than the second, third, or fourth. The distal ends of the metatarsals show evidence for movable joint connections to the phalanges as the distal ends are mediolaterally expanded and weak flexor tubercles of the phalanges slightly lap metatarsals I–IV on both sides. As is typical for all post-Triassic turtles, the fifth metatarsal is developed as a plated element with a morphology unlike those of the other metatarsals. It projects medially from the remainder of the foot and has a concave outer, medial margin, and a gently hooked distal process. In the region of this process, the first phalanx of the fifth digit articulates. This first phalanx resembles a regular metatarsal, is mediolaterally aligned with metatarsals I–IV and probably fulfilled a function equivalent to these first metatarsals. Unlike in the true metatarsals, the distal articular surface of the ‘metatarsalized’ first phalanx of the fifth digit is not overlapped by a flexor tubercle. However, interphalangeal movability is indicated by the clear presence of flexor grooves on the ventral surface, which probably served as a ligament attachment in life. The phalangeal formula of the pes is 2-3-3-3-4, whereby the ultimate phalanx on each digit is developed as an ungual, with exception perhaps of the fifth (see below). The non-ungual phalanges of digits I–IV are relatively robust, and all phalanges on all digits seem to retain movable interphalangeal articulations. In digits II–III, the first and second phalanges are each approximately equally long, but the phalanges are successively longer counted from digits I through IV. Thus, the fourth digit is the longest in each foot. In the fifth digit, the phalanges are somewhat slenderer than in preceding digits, and become shorter distally within the digit. The unguals are robust and relatively broad in digits I–III, but become slenderer in successive digits. In digit V, the ungual is miniaturized and only about half as long as the unguals in the other digits. It is unclear if this ungual on the fifth digit indeed carried a claw (and thus indeed is an ungual), or if the element ended blindly in tissue.

#### Soft tissues

NKMB Watt18/211 preserves soft-tissues in the form of a thin film of phosphatic transformation products that surround parts of the skeleton. Under UV light, these phosphatic soft tissue remains can easily be distinguished from the surrounding limestone matrix and phosphatic bone (Fig 3). In many parts of the body, including the neck and tail and parts of the limbs, the apparent contour of the animal closely mirrors the known distribution of soft-tissue. This effect is emphasized by the choice of preparation, as a wall of unremoved sediments was retained wherever the soft tissue ends. In other parts of the body, however, the preparator interpolated the known distribution of soft tissue, thereby creating the illusion of crisp margins. The shadow of soft tissue surrounding the body must therefore be viewed with caution. The soft tissue is particularly well preserved in the right forelimb. Digits I to III are clearly surrounded by soft-tissue to the bases of the unguals, but the tips of the claw remain free. Small patches of white material suggest that the remaining fingers were also enveloped by soft-tissue, but it is unclear if the claws were free. As a novelty, a broad flap of tissue is apparent behind the forelimb, which suggests that the flipper was broadened significantly by a flexible, trailing margin, as seen in extant marine turtles [45]. Patches of soft tissue found between the digits of the foot once again fully encased by soft-tissue, but the exact outline of the foot is unclear, except perhaps for the soft-tissue between digit I and II of the right pes, which suggests again that the integument encompassed the base of the unguals, but not the claws. The wavy outline of the paddle seen on both feet is a preparation artifact that should be ignored. The presence of epibionts on both feet (see below) suggests that the soft-tissue between the digits was not flexible, like the webbing of extant riverine turtles, but rather stiffened to a paddle, as in extant marine turtles (see Discussion below). Among fossil turtles, we are only aware of similarly well-preserved soft tissue in the Early Cretaceous protostegid *Rhinochelys nammourensis* [29] and the Paleogene stem cheloniid *Tasbacka* "*danica*" [46].

#### Cololite

An elongate object can be seen through the central plastral fontanelle of NKMB Watt18/211. Its irregular, wavy outline combined with its phosphatic preservation is typical of a coprolite, but its location within the visceral cavity suggests that it is a cololite (= cololith) instead. Its location midbody combined with its relatively narrow diameter is broadly consistent with the small intestine of extant marine turtles [45].

#### Epibionts

Yet another oddity of NKMB Watt18/211 is the presence of two shells between the fourth and fifth digits of each foot (Fig 3D and 3F). The shells are patelliform elements preserved with the convex side showing downwards on the left foot, but the concave side showing downwards on the right foot. Assuming that these are epibionts, this suggests that the shell on the left foot occupied the bottom side of the foot, but the shell on the right the top side of the foot. The shell preserved on the left foot shows concentric growth annuli on its external surface that suggests an elongate shell shape. The dimensions of the element preserved on the right foot, however, are unclear. The shells are preserved solidly and in three-dimensional solid condition and thus differ in this respect from other molluscan shells found in the vicinity. The excavations carried out by NKMB at Wattendorf are extremely rigorous, as all exposed sedimentary surfaces are checked for fossils, but no other examples of such shells have been found. We are therefore confident that both shells are true epibionts, not shells that coincidentally came to rest at these locations. Among epibionts described from the Jurassic, these resemble the juvenile phases of oysters the most, which are typically found attached to ammonites [47,48]. We are unaware of epibionts being preserved on any other turtle from the Late Jurassic platy limestones of Europe. Although it is possible that an unknown species of oyster specialized in colonizing this exact species of thalassochelydian turtle, the juvenile nature of the preserved specimens suggests that these animals only accidentally colonized turtles, but were shed before reaching maturity.

### Phylogenetic results

The topological results from our phylogenetic runs are identical regardless of whether "NKMB Watt18/211" or "*Thalassemys bruntrutana*" (which additionally includes information from other referred specimens) was used as the OTU. Using NKMB Watt18/211 as the only source of information resulted in 5940 MPTs with a length of 1739 steps. The same number of MPTs was found when using "*Thalassemys bruntrutana*," but these trees require an additional four character transitions, resulting in a tree length of 1743.

*Thalassemys bruntrutana* is recovered as a thalassochelydian in our analysis (Fig 4, S4 Appendix). Although this result was expected, we note that no species of *Thalassemys* had previously been included in any formal phylogenetic analysis. As our primary goal was to investigate the phylogenetic position of *T*. *bruntrutana*, we limit ourselves to reporting the most apparent differences with previous results [27] and providing only brief comments.

Whereas Evers et al. (2019) [27] recovered thalassochelydians in a poorly-supported sister-group relationship with pleurodires, we herein find thalassochelydians to be stem turtles in a more crownward position than paracryptodires, but one node more stemwardly than xinjiangchelyids (Fig 4). Although a position on the turtle stem had previously been found for thalassochelydians (e.g. [49]), their stemward position with regard to xinjiangchelyids is usually only found in studies that recover all of these turtles as stem cryptodires (e.g. [50–53]). Our tree otherwise differs in the contents and placement of the clades *Sinemydidae* and ’Macrobaenidae.’

As with regard to the ingroup relationships of *Thalassochelydia*, we find two primary clades within thalassochelydians (Fig 4). The first is *Sandownidae*, which includes *Solnhofia parsonsi*, similar to the result of [54], but contrary to the topology [27]. This result is interesting, as some of us recently presented osteological evidence suggesting that *S*. *parsonsi* may indeed be a sandownid [55], but we did not include any of these new observations into the current matrix. Instead, the positional change of *S*. *parsonsi* to the base of *Sandownidae* must be caused by the character polarization of existing characters at the base of *Thalassochelydia*, itself caused by the early-branching position of *Thalassemys bruntrutana*. This indicates that the sandownid affinity of *S*. *parsonsi* can even be found using existing character concepts and without recent suggested, but not yet integrated characters [55]. The second primary clade of *Thalassochelydia* lacks a formally defined clade name [13], but consists of taxa commonly labeled as plesiochelyids [1]. The early branching position of *Thalassemys bruntrutana* within *Thalassochelydia* should be interpreted with caution. The remaining thalassochelydians are supported by a number of cranial and mandibular synapomorphies (see S5 Appendix), many of which could not be positively scored for *Thalassemys bruntrutana*. As such, the position of *Thalassemys bruntrutana* may change with the addition of more completely scored cranial and mandibular characters.

*Thalassemys bruntrutana* shows overall larger similarity in the character scoring to chelonioids, and particularly protostegids, than most other thalassochelydians (particularly plesiochelyids) that are included in our matrix. This is for instance evident with regard to the epiplastral shape, the likely retainment of a posterior plastral fontanelle, or a reduced hypoplastral contact with the costals along the inguinal buttress. Additionally, the thalassochelydian and protostegid flippers show some similarity not shared with crown-group chelonioids, particularly that the fourth finger is longer than the third, or that the first two digits retain inter-phalangeal movability. Despite these character observations, protostegids in our analysis remain in their stem-chelonioid position [27], contradicting hypotheses that suggest protostegids and thalassochelydians may form a clade to the exclusion of chelonioids (e.g. [40,56]). Given that other groups, such as sinemydids and macrobaenids, are relatively viable in their global position in the context of our matrix in comparison to [27], and given the aforementioned character similarities between protostegids and *T*. *bruntrutana*, it may be surprising that neither protostegids are dragged stemwards toward thalassochelydians, nor that thalassochelydians are dragged crownwards into a large chelonioid total group. We think that this result can be understood as relatively strong character support within the matrix used for the respective global positions of thalassochelydians as stem turtles and protostegids as stem chelonioids, but note that contrary views based on other matrices are available (e.g., [50,53,57,58]). Nonetheless, it is clear that the current phylogenies and matrices require more improvements to provide further tests of alternative topological hypotheses. For instance, additional important taxa need to be added in the future, such as the macrobaenids *Macrobaena mongolica*, the eurysternid *Eurysternum wagleri*, the sandownid *Solnhofia brachyrhyncha*, as well as additional thalassochelydian taxa.

## Discussion

### Alpha taxonomy

#### JME 3995 –*Thalassochelydia* indet

The vast majority of named fossil turtles from the Late Jurassic of Europe are based on shells or crania [1]. It is therefore particularly challenging to refer JME 3995 to any particular taxon. Although highly fragmentary, meaningful remains of the carapace nevertheless are present, in particular a segment consisting of parts of left peripherals II–V and the lateral portions of left costals I and II (see Supporting Information). Three characteristics are notable: the narrow development of the peripherals, the well-developed fontanelles framed by the elongated free ends of the ribs, and a distance between costal rib I and II of approximately 3.6 cm. The ratio between the distance between costal rib I and II to the midline length of the carapace of coeval thalassochelydians is about 10 percent (10.3% in NKMB Watt15/1 *Achelonia formosa*; 10.5% in BSPG 1960 VIII 43 *Eurysternum wagleri*; 10.1% in MB R2441 *Solnhofia parsonsi*). Assuming similar proportions, this implies a midline carapacial length of 36cm for JME 3995. A number of thalassochelydians from the Late Jurassic of Europe are known from shells that are equal in length or smaller, in particular *Idiochelys fitzingeri* [8,16], *Solnhofia parsonsi* [59], *Tropidemys seebachi* [26], and *Parachelys eichstättensis* [17], medium-sized individuals of *Eurysternum wagleri* [60], and medium sized individuals of *Plesiochelys bigleri* Püntener et al. 2017 [61] and *Plesiochelys etalloni* Pictet and Humbert, 1857 [30,39]. In all of these, the peripherals are broad and carapacial fontanelles are reduced to absent. Large specimens are known for *Craspedochelys jaccardi* Pictet, 1860 [39,62] and *Tropidemys langii* Rütimeyer, 1873 [8,63], but these lack any trace of anterior plastral fontanelles as well. As plastral fontanelles close during ontogeny, we conclude that JME 3995 cannot be referred to any of these taxa. The pes is rarely preserved in Late Jurassic turtles.

Three turtle species are known from specimens equal or larger than JME 3995 that still display enlarged plastral fontanelles: *Pelobatochelys* (or *Tropidemys*) *blakii* Seeley 1869 [64,65], *Achelonia formosa* [6] (= *Enaliochelys chelonia* Seeley 1869 [26,28,64]), and medium sized individuals of *Thalassemys bruntrutana* [19,28]. To date, none of these have been reported from Tithonian deposits, but the large bodied *Owadowia borsukbialynickae* [66], which shows similarities with *Thalassemys* spp. (see below), suggests that large bodied turtles are present during the early Tithonian as well. We therefore cautiously conclude that JME 3995 represents a large bodied thalassochelydian with possible affinities with *Thalassemys* spp.

#### NKMB Watt18/211 –*Thalassemys bruntrutana*

At present, only four species of large bodied thalassochelydians (i.e., carapace length greater than 60cm) are known from Late Jurassic deposits throughout Europe: *Achelonia formosa* (= *Enaliochelys chelonia*), *Pelobatochelys* (also *Tropidemys*) *blakii*, *Thalassemys bruntrutana*, and *Thalassemys hugii* [1,26,28,37]. NKMB Watt18/211 resembles *Achelonia formosa* by its large size and by having an overall rounded carapacial outline, but differs by having relatively small carapacial and plastral fontanelles, despite being considerably larger in size, in contrast to the shell of *Achelonia formosa*, which possess large plastral central and lateral fontanelles and well-developed carapacial fontanelles anterior and posterior to each costal rib [26,28]. At present little is known about *Pelobatochelys* (also *Tropidemys*) *blakii*, but NKMB Watt18/211 differs, as before, by lacking extensive carapacial fontanelles and, most notable, by lacking a low middorsal keel [28]. NKMB Watt18/211 resembles *Thalassemys hugii* and *Thalassemys bruntrutana* by its large size, and by generally lacking carapacial and lateral plastral fontanelles, but differs from *Thalassemys hugii*, but resembles *Thalassemys bruntrutana*, by having a broader shell and by lacking confluent central plastral and xiphiplastral fontanelles [19,39]. All previously described specimens of *Thalassemys bruntrutana* have been described from the late Kimmeridgian of Europe [1,19,28]. Considering the apparent overlaps in morphology and time, we confidently refer NKMB Watt18/211 to *Thalassemys bruntrutana*. This greatly expands the known morphology of this taxon to include aspects of the cranium, vertebral column, the girdles, and limbs, and aspects of the shell, in particular the epiplastron and peripheral series. The biogeographic distribution is furthermore expanded from Switzerland [19] and the United Kingdom [19,28] to now include southern Germany as well.

***Owadowia borsukbialynickae***. Szczygielski et al. (2018) [66] recently described a new fossil turtle from the early Tithonian of Sławno, Poland for which they coined the name *Owadowia borsukbialynickae*. Although the type material only consists of a partial mandible, right coracoid, right ilium, and partial right femur, it is notable among early Tithonian faunas for its large size. The species was differentiated relative to other Late Jurassic turtles, among others, by the elongate and spatulate shape of the mandibular symphysis and the low angle between the mandibular rami [66], but its validity is tenuous, as the majority of European turtles from this time period are not known by this body part, particularly the larger species. Although comparisons are limited, we note that the mandibular symphysis of NKMB Watt18/211 and *Owadowia borsukbialynickae* broadly correspond to one another by being elongate and spatulate and by having a similar angle. In addition, the portion of the dentary that bears the ramphotheca is swollen relative to the rest of the mandible in both as well. Although the type of the early Tithonian *Owadowia borsukbialynickae* is far too incomplete to allow further comparison with published late Kimmeridgian specimens of *Thalassemys hugii* and *Thalassemys bruntrutana*, the above listed similarities suggest close proximity. We therefore foresee that future finds may either lead to the synonymization of *Owadowia borsukbialynickae* with one of two already named species of *Thalassemys*, or the referral of *O*. *borsukbialynickae* to *Thalassemys*.

### Flippers

#### Evidence for marine adaptation

The relative length of the hand of extant turtles is known to correlate well with their habitat, in that turtles with long hands are more aquatic than those with short hands [11], but an elongate flipper by itself is not sufficient to postulate marine versus freshwater aquatic preferences. This is most apparent by reference to the extant *Carettochelys insculpta*, which possesses hands that resemble those of extant chelonioids in their proportions [11], and function in underwater "flight" [67], but nevertheless lives in rivers [43]. The flippers of *Carettochelys insculpta* even resemble those of marine turtles in many structural details, including the permanent hyperextension of the elbow, the reduction in the number of claws, and the fusion of the phalanges in some digits [68]. Given that the turtles from the platy limestones of Europe were likely washed into the basins from a broad set of habitats [12], we are therefore left to wonder if *Thalassemys bruntrutana* utilized its elongate forelimbs (Fig 5) to navigate rivers or oceans.

The elongate forelimbs of extant riverine and marine turtles differ in numerous structural details, but few of these can be postulated to be necessarily related to ecology. We here argue that the skin that covers the limbs may be an exception. In general, turtles, like all reptiles, are able to form scales that cover their body. While much literature is devoted to documenting variation to the scales (= scutes) that cover the shell [69], the enormous morphological variability that is apparent in the presence, size, and distribution of scales over the remainder of the body (Fig 6) is mostly undocumented beyond occasional observations pertaining to cranial scalation or hold-fasts utilized in mating [43]. The epidermis of turtles serves as an important barrier in preventing water loss, either due to osmotic drying in marine water or evaporative drying on land [70–74]. While the neck, tail, and limbs of turtles are primarily covered by flexible alpha keratin, these areas can also be covered by scales that may include water-impermeable β-keratin [75]. A greater cover of the body with scales therefore plausibly correlates with greater protection against water loss. This correlation appears to be supported by qualitative observations we undertook on a large sample of extant turtles. Forms that rarely leave their freshwater aquatic habitat, in particular carettochelyids, trionychids, chelydroids, but also riverine pleurodires and testudinoids, tend to have limbs devoid of scales, including the extensive webbing that spans between the digits (Fig 6B). The limbs of pond turtles that often venture onto land, including many pleurodires and testudinoids, are covered by fine scales, but the webbing still remains free of scales (Fig 6C), likely to maintain their function in rowing. The skin of the most terrestrially adapted turtles, only seen in terrestrial testudinoids, is systematically covered by thick, pyramidal scales that are often underlain by bone (Fig 6A). Finally, the limbs of marine turtles, are systematically covered by scales, of which smaller ones aid in the movement of the limb, while large ones stiffen the paddles (Fig 6D). Although the apparent changes to scalation are gradual (and not strictly linear) along the spectrum from riverine to terrestrial turtles, a stark difference is apparent between long-handed riverine and long-handed marine turtles, as the flippers of marine turtles are fully covered by scales, which we interpret as a necessary prerequisite for life in the ocean, in contrast to riverine turtles, which show poor to no scalation. The absence of scales in riverine turtles may be an evolutionary compromise, as the resulting, flexible hands are not optimal for generating propulsion, at least when compared to marine turtles [76], but allow for other functions, such as the manipulation of food, gripping logs while sleeping, climbing onto land for basking, or walking on land. An important exception that breaks the correlation we establish above is the extant pelagic leatherback turtle, *Dermochelys coriacea*, which as an adult is fully covered by a thick, leathery skin that superficially resembles that of marine mammals [43], but the presence of scales in juveniles suggests to us that this is a secondary adaptation to marine life, not a necessary primary adaptation.

**Fig 7:**
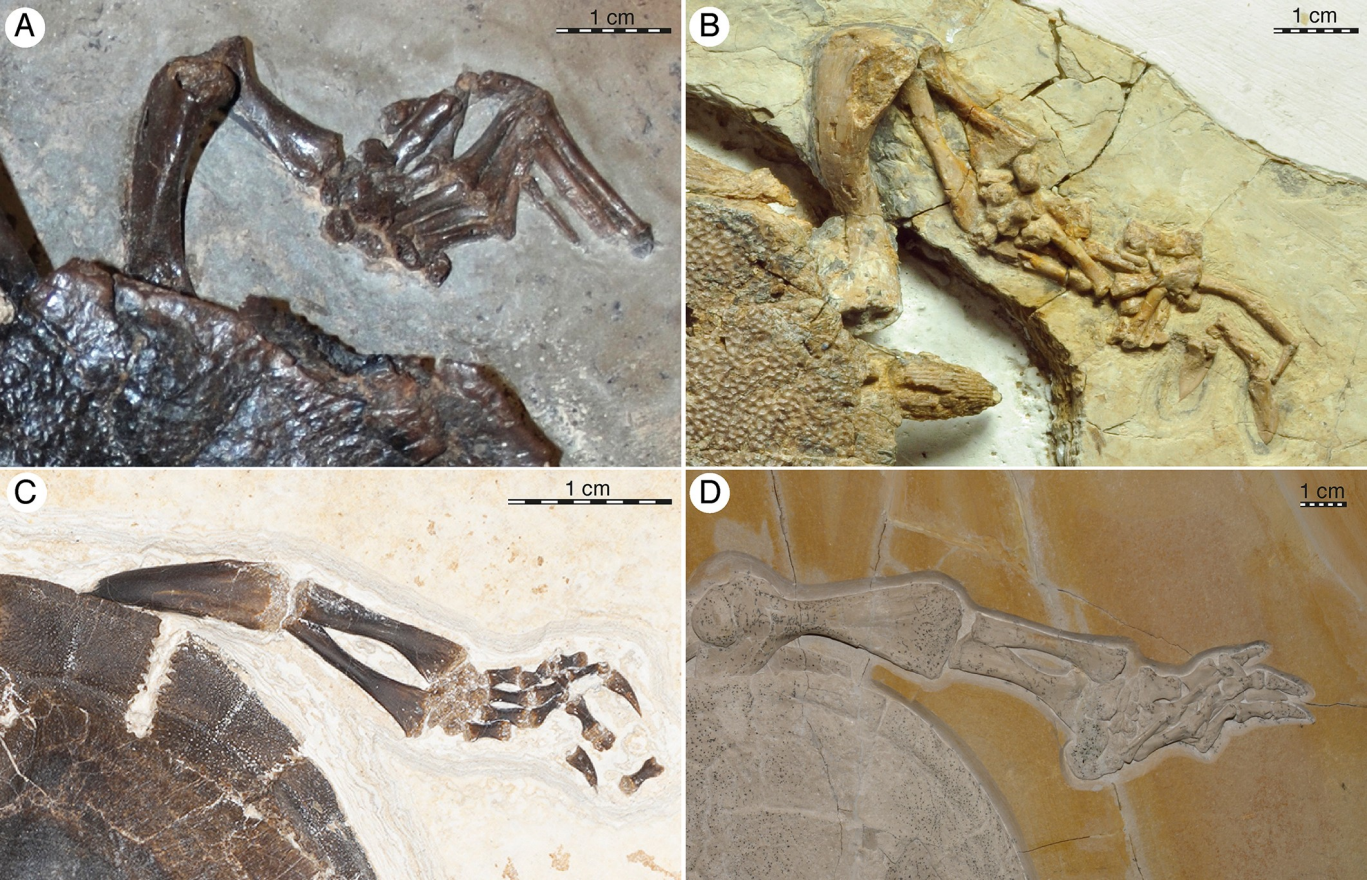
Taphonomic preservation of freshwater adapted turtle forelimbs in dorsal view. (A) the carettochelyid *Allaeochelys crassesculpta* from the Eocene Messel Formation of Germany (IRSNB AFR34). (B) the pan-trionychid *Perochelys lamadongensis* from the early Cretaceous Jehol Biota of China (IVPP V 18048). (C) the testudinoid turtle *Echmatemys* sp. (FOBU 14014) from the Green River Formation of the USA. (D) *Parachelys* sp. (NKMB Watt05/202) from the Late Jurassic of Wattendorf, Germany. Note that the forelimb is slightly adducted in all cases and that the digits are stacked on top of one another. The images are not to scale.

**Fig 8:**
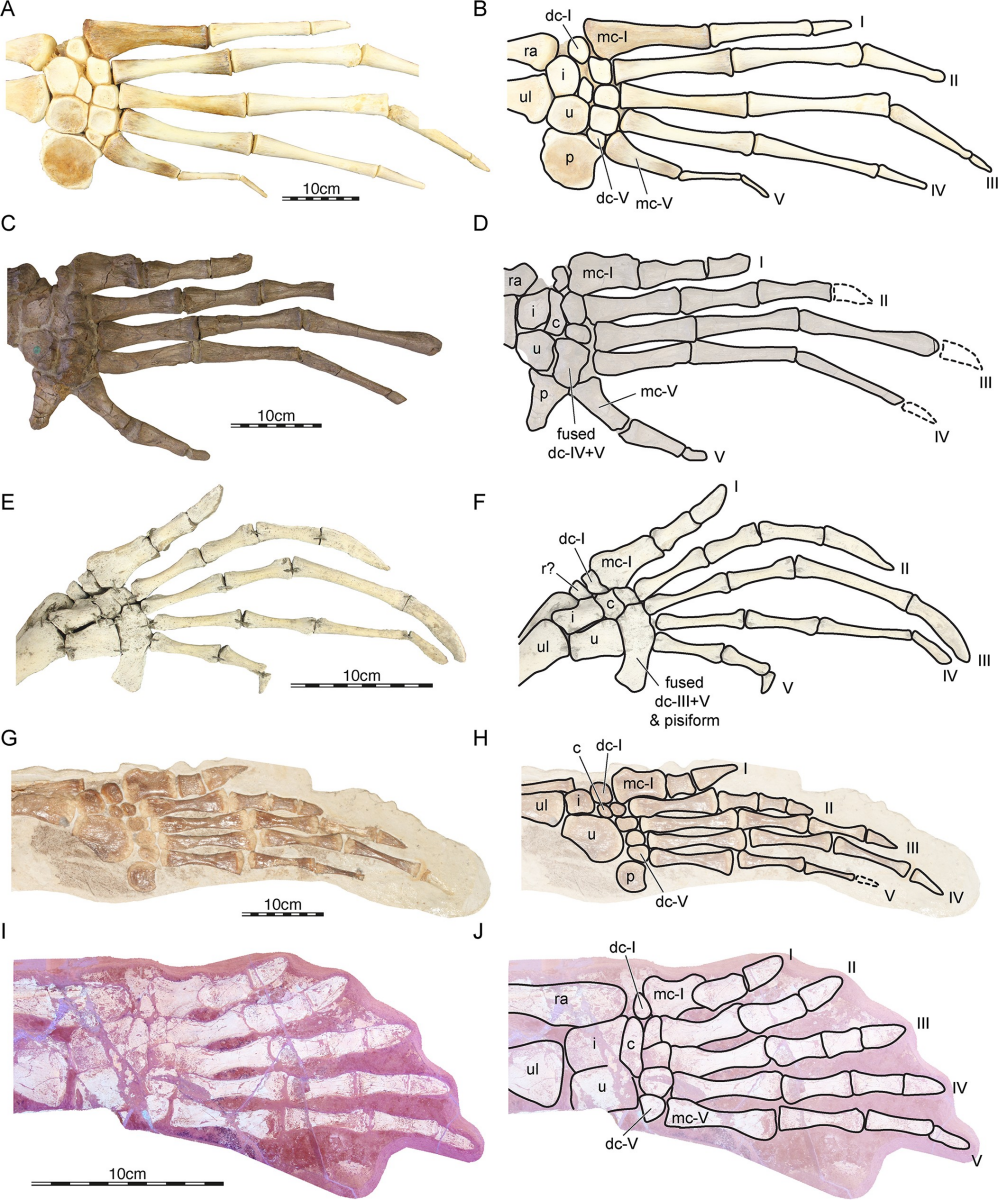
Comparisons of forelimbs of marine adapted turtles. **(**A) photograph and (B) line drawing of reflected left flipper of the extant leatherback turtle *Dermochelys coriacea* (QM J47453) in dorsal view. (C) photograph and (D) line drawing of right flipper of the extinct chelonioid *Allopleuron hofmanni* (NHMUK PV 42893) in dorsal view. (E) photograph and (F) line drawing of the right flipper of the extant cheloniid *Natator depressus* (QM J14463) in dorsal view. (G) photograph and (H) line drawing of right flipper of the extinct protostegid *Rhinochelys nammourensis* (MSNM V3933; picture courtesy of Ren Hirayama) in dorsal view. (I) photograph and (J) line drawing of reflected left flipper of the extinct thalassochelydian *Thalassemys bruntrutana* (NKMB Watt18/211) in ventral view. Roman numerals denote digit identity. Abbreviations: c, central carpal; dc, distal carpal; i, intermedium; mc, metacarpal; p, pisiform; r, radiale; ra, radius; u, ulnare; ul, ulna.

Although we are not able to conclude forcefully that JME 3995, an isolated hind limb, can be referred to *Thalassemys*, the unique preservation of a paddle-like flipper in this specimen stiffened by enlarged scales (Fig 1) is highly suggestive that marine adapted animals were present in the early Tithonian platy limestones of southern Germany. The convergence with the marine adapted paddle of extant cheloniids is striking, in particular the stabilization with scales of an expanded trailing edge. Although no scales are preserved in NKMB Watt18/211 (Figs 2 and 3), three observations allow us to conclude that the soft-tissue preserved between the digits is not the flexible webbing found in riverine turtles, but rather the stiffened paddles of marine turtles. First, the pes of NKMB Watt18/211 corresponds in all details to that of JME 3995, including the development of trailing edges to the paddles, which is otherwise only seen among extant marine turtles (Fig 6). Second, the presence of epibionts in the interdigital space of both feet suggests strongly that the pes forms a stiffened paddle covered by large scales. Although epibionts are known to colonize extant leatherback turtles [77], including small species specialized on colonizing the flexible, but smooth flippers (pers. comm. Nathan Jack Robinson via Eric Lazo-Wasem 2021), we find it unlikely that the juvenile oysters found on NKMB Watt18/211 would colonize smooth skin, given that they otherwise are known to settle on hard shells, such as those of ammonites [47,48]. Finally, the manus and pes of NKMB Watt18/211 are perfectly splayed out in a pose consistent with the development of stiffened flippers. Although this final observation may seem to be a weak argument, as the limbs of riverine turtle may feasibly come to rest in this position as well, our survey of fossilized turtles suggests that the limbs of riverine turtles typically come to rest slightly adducted and with the digits of the limbs stacks on top of each other, particular in formations with laminar sediments. Examples are available from the Early Cretaceous Jehol Biota in China (Fig 7A [78–81]), the Eocene Messel Formation in Germany (Fig 7B; [82]) and the Eocene Green River Formation in the USA (Fig 7C; [83]). In contrast, the stiffened flippers of unambiguous marine turtles typically come to rest in a pose that resembles NKMB Watt18/211 (Figs 2 and 3). Examples are available from the Late Cretaceous Sannine Formation of Lebanon (Fig 8G; [29]) and the Paleocene Fur Formation of Denmark [46]. Turtles from the Late Jurassic platy limestones of Europe show both poses (Fig 7D), which supports the notion that both marine and freshwater aquatic turtles are deposited in the basins. The conclusion that *Thalassemys bruntrutana* is a marine turtle is supported by a series of accessory observations, none of which are not conclusive by themselves, but consistent nonetheless. These include the relatively large body size, the presence of extensive plastral fontanelles, and likely pronounced sexual dimorphism in tail length.

Late Jurassic marine deposits across Europe regularly yield large-bodied turtles (see [1] for recent summary), but this is the first study that demonstrates positively that at least one of them, *Thalassemys bruntrutana*, was adapted to permanent life in the ocean. This further underlines the recent taxonomic conclusion, that this large-bodied species occurs across the epicontinental seaways that crossed continent, not just particular basins [19,26,37,84]. The marine adapted flippers in combination with its occurrence across Europe suggest that *Thalassemys bruntrutana* was able to navigate the open ocean, which in return raises the question why thalassochelydians are restricted to Europe, with the sole exception of *Neusticemys neuquina*, which is known from the Tithonian Vaca Muerta Formation of Argentina [85,86]. As the Late Jurassic seaways of Europe were broadly connected to the Arctic Ocean, North Atlantic, and Tethys and as collecting efforts were historically focused on the Late Jurassic of Europe and Argentina, we predict that the group will be found in additional places outside of Europe in the future.

*Convergence in marine turtle flipper evolution*. The novel anatomical characterization of a thalassochelydian flipper allows us to study the independent morphological evolution of flippers in two secondarily marine turtle clades (Fig 8) and to comment on convergences and differences. The evolution of the pan-chelonioid flipper suggests an early burst of character acquisition at the base of the total group (*Pan-Chelonioidea*, including *Toxochelys* and protostegids), and subsequent minor changes in individual lineages (e.g. protostegids, dermochelyids, cheloniids, ctenocheyids [27]). Early skeletal modifications in pan-chelonioids are those that likely positively affect the swimming biomechanics (flattening of elements; relative lengthening of the humerus; stiffening of finger elements) and movability of the arm (anterior radius position; lateral humeral process position; widening of the internal angle of the scapula), and are thus herein labeled as ‘fundamental’ flipper traits [27]. This is in accordance with some of these adaptations, particularly the flattening of carpal and arm elements, modifications to the elbow joint, and the relative lengthening of the humerus, which are widespread among secondarily marine tetrapods in generally [87]. As these traits are shared among turtles between protostegids, *Toxochelys* (which only lacks the wide internal scapular angle), and non-protostegid pan-chelonioids [27], a possible phylogenetic position of protostegids outside of the chelonioid total-group (e.g. [50]) would not affect these flipper innovations at the base of *Pan-Chelonoidea*, but simply require their independent acquisition by *Protostegidae*. Although our new phylogeny suggests the additional inclusion of ‘macrobeanids’ as the earliest branching member of *Pan-Chelonioidea*, this does not affect the sequence of flipper character acquisitions in deeper nested nodes of plausibly marine pan-chelonioids (i.e. *Toxochelys*, protostegids, crown chelonioids).

*Thalassemys bruntrutana* shows the majority of ‘fundamental’ flipper traits also seen in pan-chelonioids (Fig 8), indicating that these characters evolved convergently and underlining their importance as potential functional requirements for flipper-aided swimming in turtles. In particular, the radius is positioned anteriorly to the ulna, resulting in the hyperextension of the elbow (ch. 339.1), the outer fingers lack clear interdigital articulations suggesting they are stiffened (ch. 342.1; Fig 8), the pedal and manual elements are flattened (ch. 350.1; Fig 8), and the internal scapular angle exceeds 110° (ch. 313.1). Notably, the highly efficient freshwater aquatic turtle *Carettochelys insculpta* also shows an elbow hyperextension via an anterior radius displacement [68], which is not seen in closely related trionychids, themselves able swimmers. This suggests that at least the anterior radius displacement and elbow hyperextension is directly linked to the subaqueous ‘flying’ observed in extant chelonioids and *Carettochelys*, and further suggests that fossil groups with the same anatomy (e.g. protostegids, *T*. *bruntrutana*) also employed this style of swimming. Despite these similarities to pan-chelonioids, the flipper morphology of *T*. *bruntrutana* also shows traits that suggest it is less derived, and potentially less well adapted and more primitive than the pan-chelonioid flipper. For instance, *T*. *bruntrutana* retains well-defined interphalangeal articulation surfaces on the first two (and possibly also the third) manual digits (ch. 341.0; Fig 8), suggesting the hand was not entirely stiffened along the finger joints. This is also seen in *Toxochelys*, which is unambiguously regarded as an early branching stem-chelonioid (e.g. [49,50,57,58,88,89]), and possibly some early protostegids [44], although protostegid flipper morphology needs re-evaluation regarding this feature (see [27]). Another intermediate trait is the lateral process of the humerus of *T*. *bruntrutana*, which is displaced from the proximal end of the bone, but less strongly so (ch. 332.1) than in pan-chelonioids (ch. 332.2). Examples of plesiomorphic character retention are that, unlike in pan-chelonioids, the humerus remains shorter than the femur in *T*. *bruntrutana* (ch. 337.0), and, although hard to judge with certainty from the fossil, that the coracoid likely is not elongated with respect to the humerus (ch. 314.?) contrary to pan-chelonioids (ch. 314.1). The only thalassochelydian for which this character can currently be assessed with certainty is the sandownid *Leyvachelys cipadi* [90], which clearly lacks an elongated coracoid (ch. 314.0). The humerus being longer than the femur is a characteristic trait of chelonioids [27,44] and a trait common in secondarily marine tetrapods more generally (although absent in thalattosuchians and many sauropterygians) [87], and the absence of this trait in *T*. *bruntrutana* clearly speaks to a lesser degree of flipper development compared with pan-chelonioids. Despite these proportional differences to pan-chelonioids in the humerus and coracoid, *T*. *bruntrutana* does show a key trait of flipper evolution, which is proportional epipodial shortening: whereas the humerus contributes 35% and the hand 42% of the total arm length, the ulna accounts for only 23%. Added to proportional limb data [11], *T*. *bruntrutana* falls in between of trionychids and cheloniids, although closer to the former than the latter. (Fig 5). Interestingly, the reduction of relative epipodial length is one of the few traits shared universally among secondary marine tetrapods that developed flippers, whereas anatomical modifications (e.g. hyperphalangy or element flattening) vary with clades (e.g. [91,92]).

In summary, the independent flipper evolution in thalassochelydians and chelonioids reveal parallel acquisition of ‘fundamental’ flipper traits related to the biomechanics, moveability, and proportions of the arm, which likely evolved convergently as an adaptation to effective marine, flipper-aided propulsion. None the less, chelonioid flippers possess some derived features that are not apparent in *Thalassemys bruntrutana*, highlighting that some anatomical differences remain between the flippers of thalassochelydians and pan-chelonioids.

## Supporting information

S1 Appendix Supplementary images of JME 3995. (PDF)

S2 Appendix Character list with documentation of modified characters. (PDF)

S3 Appendix Character taxon matrix in nexus format. (TNT)

S4 Appendix Full results of phylogenetic analysis. (PDF)

S5 Appendix List of common synapomorphies. (TXT)
