# Supplementary material for: Two turtles with soft tissue preservation from the platy limestones of Germany provide evidence for marine flipper adaptations in Late Jurassic thalassochelydians
Source: PLoS One. 2021 Jun 3;16(6):e0252355. doi: 10.1371/journal.pone.0252355 (PMC8174742; doi:10.1371/journal.pone.0252355)
Supplement: S1 Appendix — (PDF) [file pone.0252355.s001.pdf]

S1 Appendix. Supplementary images of JME 3995

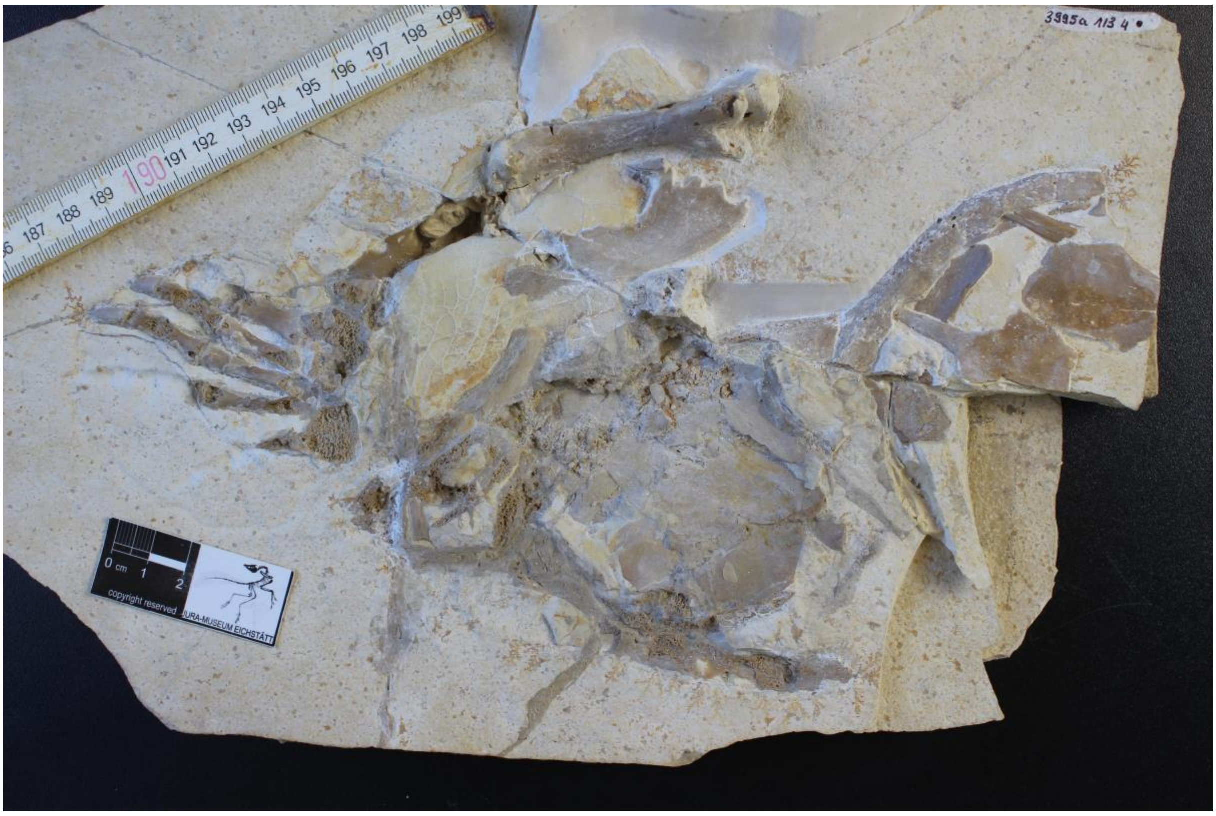

Fig S1. JME 3995, *Thalassochelydia* indet., Late Jurassic (early Tithonian) of Birkhof, Bavaria, Germany. Overview of main slab. Note the carapacial remains on the right side.

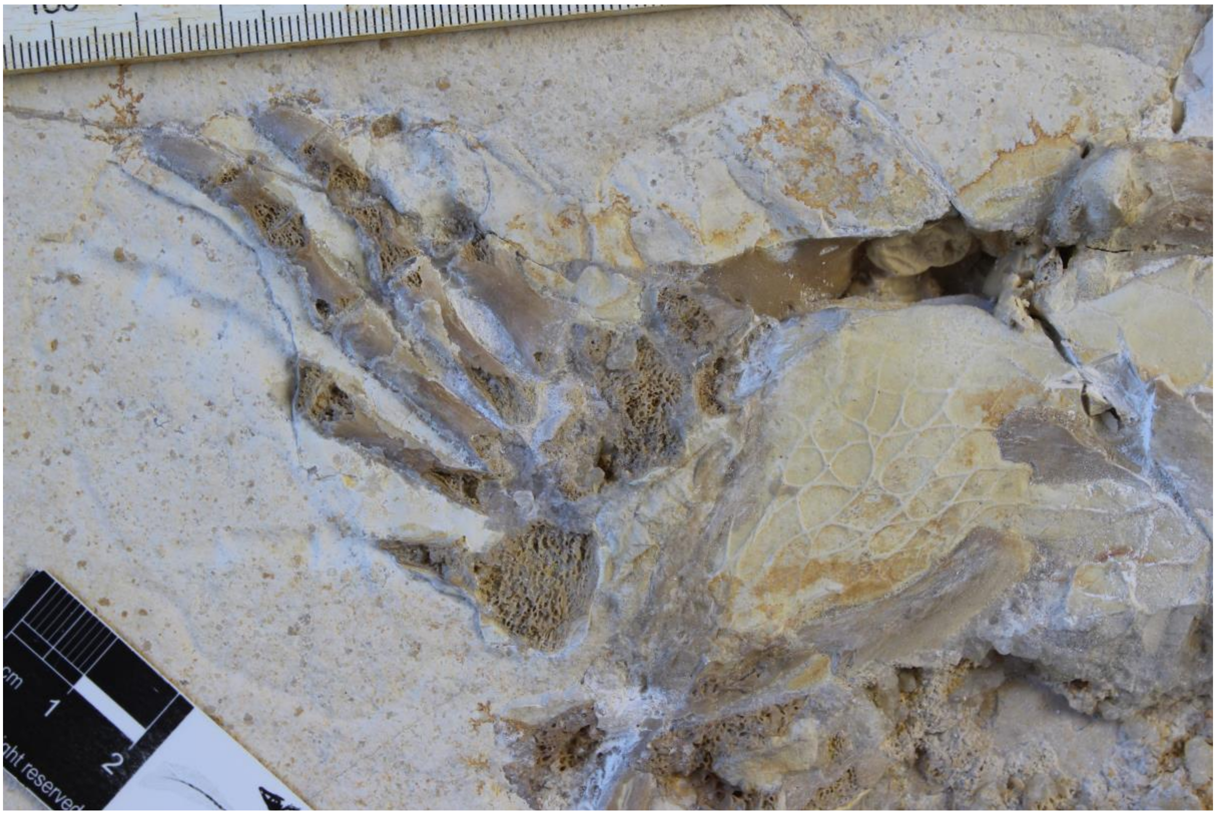

Fig S2. JME 3995, *Thalassochelydia* indet., Late Jurassic (early Tithonian) of Birkhof, Bavaria, Germany. Details of the foot on the main slab. Note particularly distinct impression of skin along the calf.

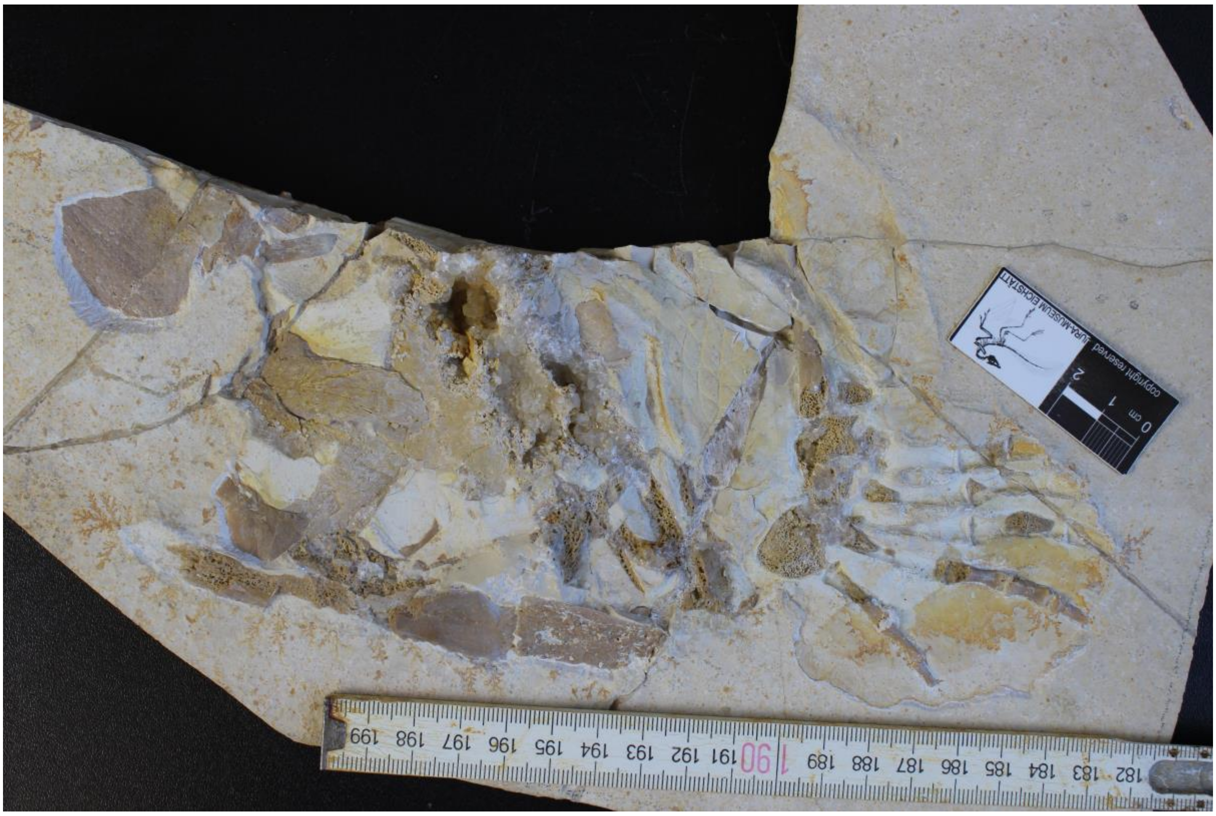

Fig S3. JME 3995, *Thalassochelydia* indet., Late Jurassic (early Tithonian) of Birkhof, Bavaria, Germany. Overview of counter slab. Note peripheral elements at the bottom.

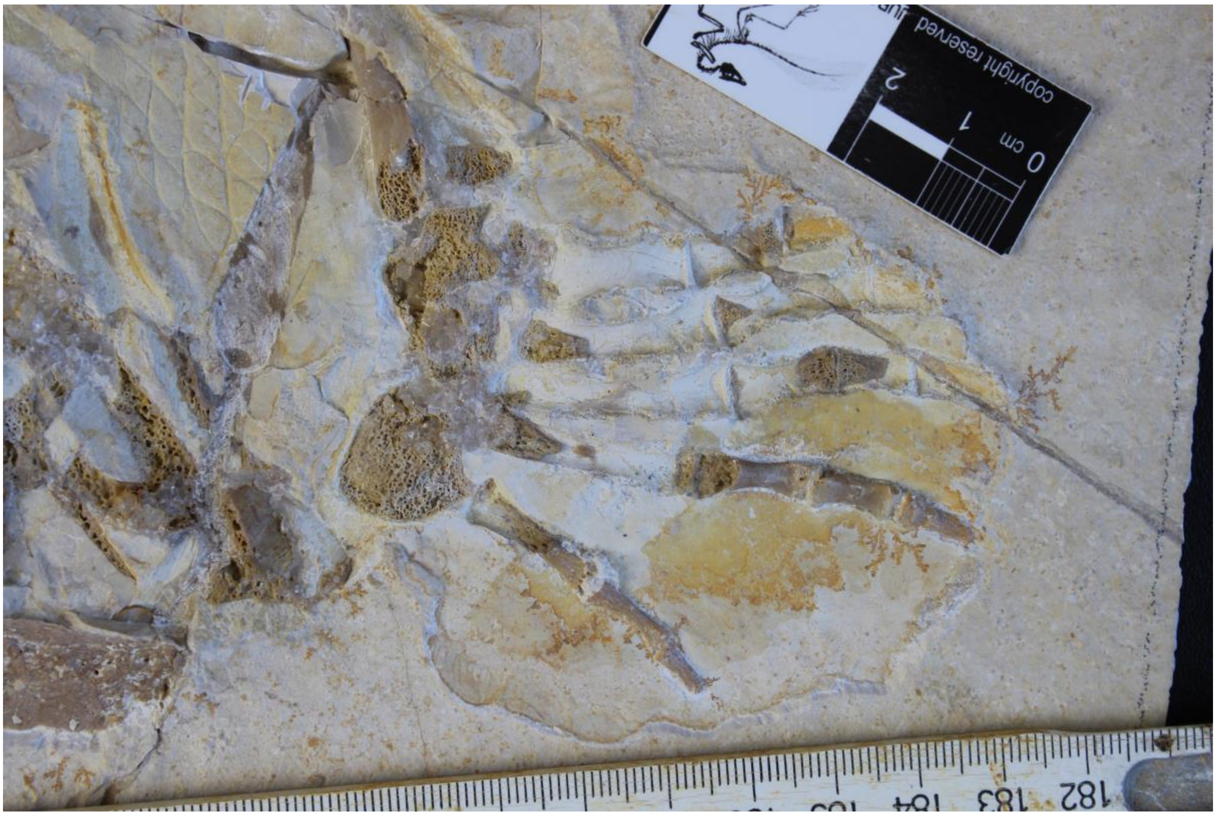

Fig S4. JME 3995, *Thalassochelydia* indet., Late Jurassic (early Tithonian) of Birkhof, Bavaria, Germany. Details of the foot on the counter slab. The outline of the foot is particularly distinct on this slab.

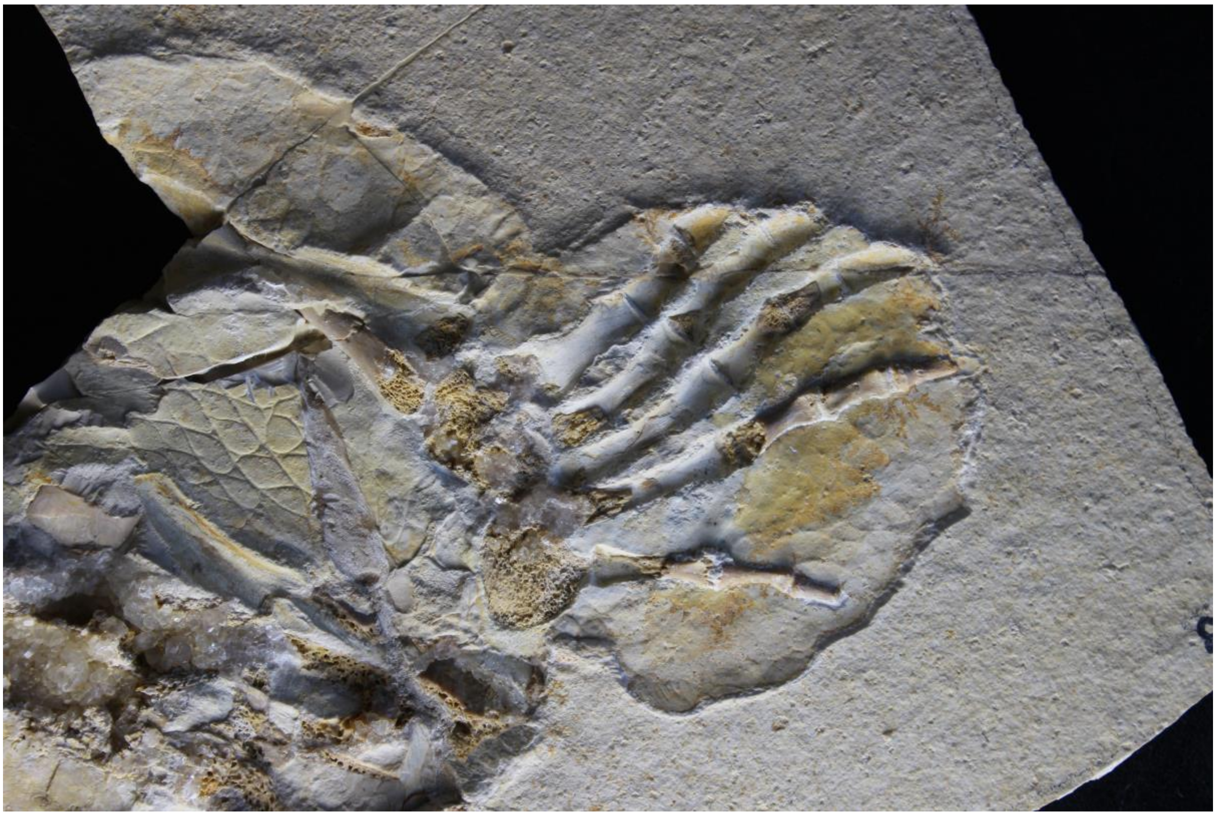

Fig S4. JME 3995, *Thalassochelydia* indet., Late Jurassic (early Tithonian) of Birkhof, Bavaria, Germany. Details of the foot on the counter slab under low light highlighting less distinct scales along the shin and foot of this animal.
