# Supplementary material for: Two turtles with soft tissue preservation from the platy limestones of Germany provide evidence for marine flipper adaptations in Late Jurassic thalassochelydians
Source: PLoS One. 2021 Jun 3;16(6):e0252355. doi: 10.1371/journal.pone.0252355 (PMC8174742; doi:10.1371/journal.pone.0252355)
Supplement: S4 Appendix — (PDF) [file pone.0252355.s004.pdf]

S4 Appendix. Full results of phylogenetic analysis

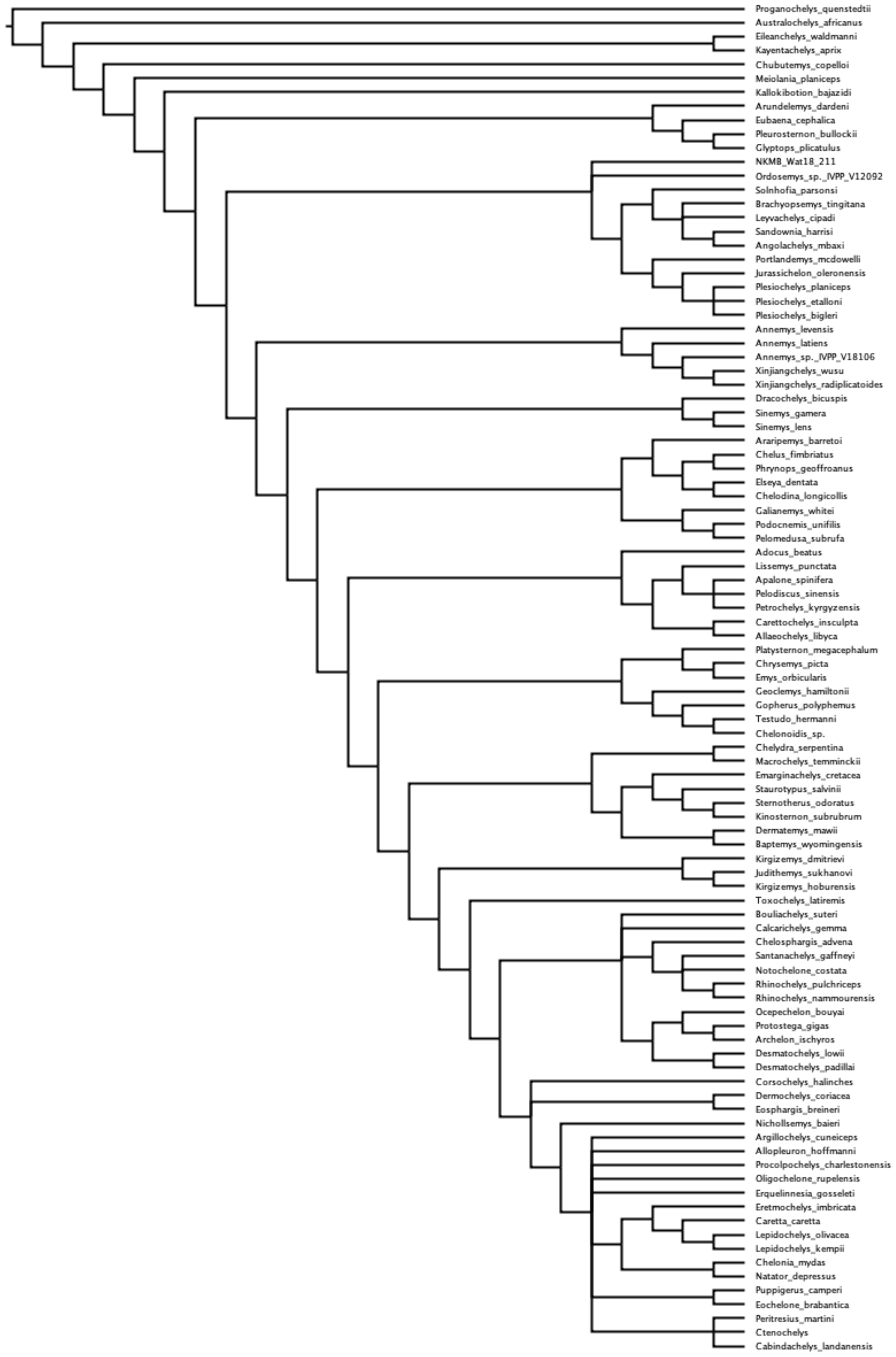

Full phylogeny using NKMB Wat18/211 as an OTU. Strict consensus topology of 5940 MPTs each 1739 steps long.

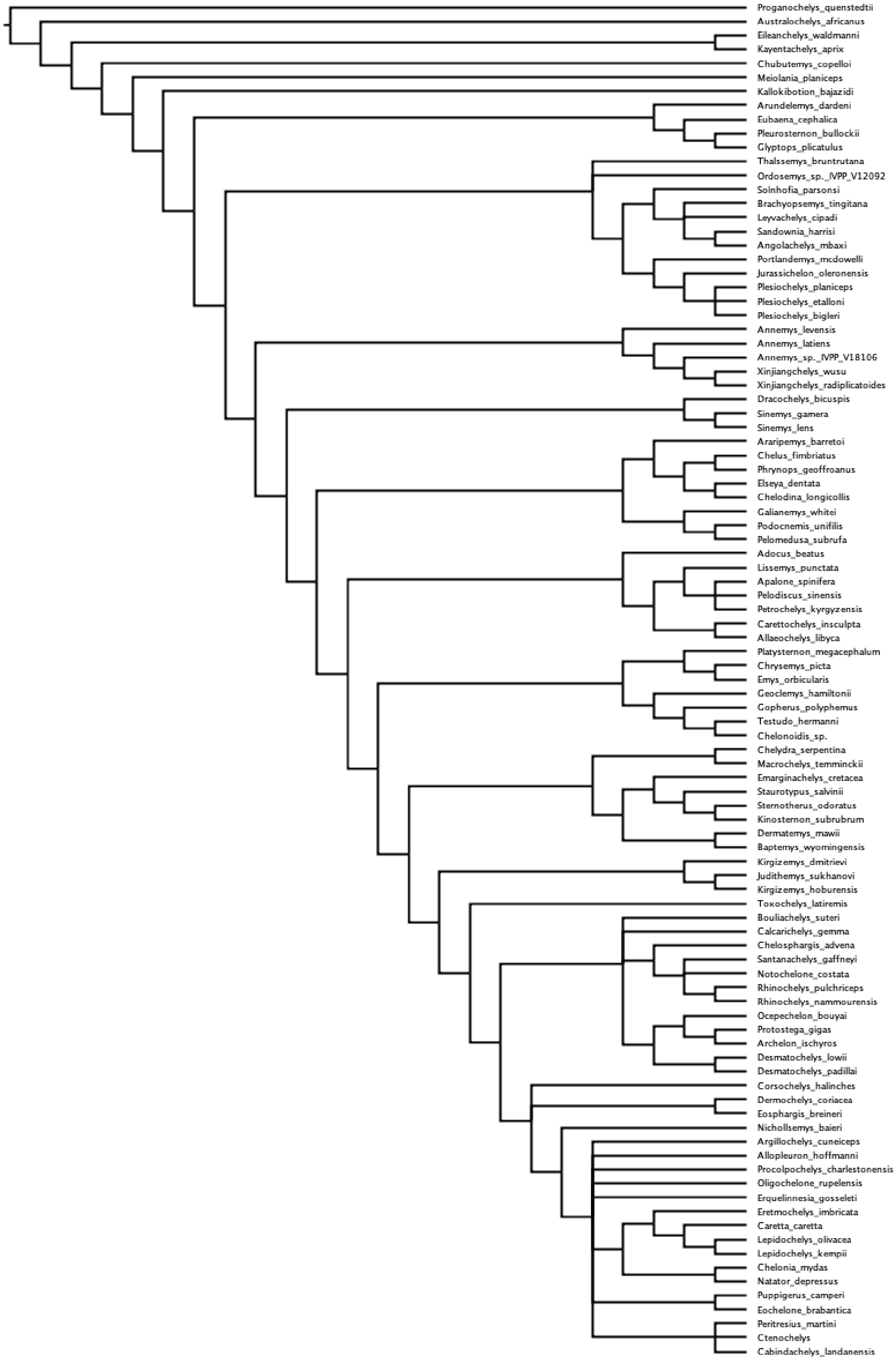

Fig S2. Full phylogeny using NKMB Wat18/211 and additional material referred to *Thalassemys bruntrutana as* an OTU. Strict consensus topology of 5490 Mpts each 1743 steps long.
